# Characterizing intra- and intertumor heterogeneity in ovarian high-grade serous carcinoma subtypes using single-cell and spatial transcriptomics

**DOI:** 10.1101/gr.281433.125

**Published:** 2026-10

**Authors:** Weishan Li, Laurie Grieshober, Jason Gertz, Adriana Ivich, Jennifer A. Doherty, Casey S. Greene, Stephanie C. Hicks

**Affiliations:** 1 Computational Bioscience Graduate Program, University of Colorado Anschutz, Aurora, Colorado 80045, USA;; 2 Huntsman Cancer Institute, University of Utah, Salt Lake City, Utah 84112, USA;; 3 Department of Population Health Sciences, University of Utah, Salt Lake City, Utah 84112, USA;; 4 Department of Oncological Sciences, University of Utah, Salt Lake City, Utah 84112, USA;; 5 Department of Biomedical Informatics, University of Colorado Anschutz, Aurora, Colorado 80045, USA;; 6 Department of Biostatistics, Johns Hopkins Bloomberg School of Public Health, Baltimore, Maryland 21205, USA;; 7 Department of Biomedical Engineering, Johns Hopkins University, Baltimore, Maryland 21218, USA;; 8 Data Science and AI Institute, Johns Hopkins University, Baltimore, Maryland 21209, USA;; 9 Kavli Neuroscience Discovery Institute, Johns Hopkins University, Baltimore, Maryland 21218, USA;; 10 Center for Computational Biology, Johns Hopkins University, Baltimore, Maryland 21218, USA;; 11 Malone Center for Engineering in Healthcare, Johns Hopkins University, Baltimore, Maryland 21218, USA

## Abstract

Ovarian high-grade serous carcinoma (HGSC) is an aggressive ovarian cancer with a heterogeneous tumor microenvironment (TME). Advances in single-cell RNA sequencing (scRNA-seq) and spatially resolved transcriptomics have enabled the study of complex TME. This study explores connections between molecular subtypes described from bulk transcriptomes and spatial domains characterized by distinct gene expression in HGSC and their variability between patients. We quantify both intra- and intertumor heterogeneity across 2D space and identify differing spatial patterns of gene expression pertaining to immune pathways and vasculature development. Functional characterization of tumor spaces reveals potentially shared cell states across molecular subtypes, whereas correlation analysis underscores subtype-specific spatial anticolocalization between spots exhibiting antigen-presenting functions and B cell–mediated immunity. Lastly, we perform spatially aware cell–cell communication analysis on the spatial samples and identify a molecular subtype specific difference in total signaling activity and heterogeneity in midkine signaling between the differentiated subtypes. Our results suggest that generating multiple tissue slices per patient might be necessary to enable comprehensive characterization of HGSC spatial transcriptomes.

Ovarian high-grade serous carcinoma (HGSC) is a common and highly aggressive type of ovarian cancer, which is generally associated with poor prognosis (Peres et al. 2019). It is characterized by the loss of *TP53* and extensive copy number alterations (Cancer Genome Atlas Research Network 2011). HGSC also has a highly complex and heterogeneous tumor microenvironment (TME) broadly encompassing: (1) the parenchyma formed by epithelial cells and (2) the tumor-associated stroma from a collection of endothelial cells, inflammatory cells, cancer-associated fibroblasts, and stem/progenitor cells. HGSC tumor progression is a product of dynamic, reciprocal interaction between the parenchyma and growth supporting stroma (Hanahan and Weinberg 2011).

Recent advances in single-cell RNA sequencing (scRNA-seq) have shed new light on the HGSC TME (Izar et al. 2020; Hippen et al. 2023). Molecular subtyping from bulk RNA sequencing (RNA-seq) provides a framework for categorizing HGSC samples based on gene expression patterns that are associated with survival and may have therapeutic implications. Tothill et al. (2008), using microarrays, were the first to report distinct molecular subtypes including the C1 subtype with strong stromal signature, the C2 subtype with elevated immune infiltration, the C3 subtype expressing a proliferative (PRO) signature, and the C5 subtype overexpressing mesenchymal (MES) genes. Subsequent studies expanded on this observation and recapitulated described subtypes, identified survival differences, and revealed that most tumors exhibited markers for more than one subtype (Cancer Genome Atlas Research Network 2011; Verhaak et al. 2012; Konecny et al. 2014). The development of consensusOV (cOV), an R/Bioconductor package that implements four major methods for HGSC molecular subtype classification as well as a random forest-based consensus classifier has facilitated generalizable and robust HGSC subtype prediction (Chen et al. 2018). Another recent advancement was the creation of a platform-agnostic, RNA-seq-compatible HGSC subtype clustering pipeline, which also demonstrated consistent clustering of HGSC samples across ethnic groups (Davidson et al. 2024).

More recently, molecular subtyping has been integrated into several HGSC gene expression atlases, enabling a deeper understanding of the role of stromal cell types and cell–cell interactions within each molecular subtype. For example, Olbrecht et al. (2021) integrated *N* = 7 HGSC scRNA-seq samples and evaluated the contribution of the stromal cell type to the *k* = 4 cOV molecular subtype signatures and identified differing cell–cell interaction. Deng et al. (2022) applied cOV to *N* = 5 HGSC scRNA-seq samples and reported subtyping results to be largely associated with cellular composition.

Furthermore, spatially resolved transcriptomics offers a new perspective to understand the cellular composition in tumors by profiling gene expression in situ, which is lost in traditional scRNA-seq techniques (Young et al. 2018). This enables profiling the tissue architecture of solid tumors and interactions between different cell types (Zheng and Fang 2022). For example, recent work spatially profiled *N* = 6 breast cancer samples and unraveled distinct cancer phenotypes, indicated by gene module enrichment scores, occupying mutually exclusive regions in space (Wu et al. 2021a). Integrating scRNA-seq results with spatially resolved data can help address the resolution limitation of nontargeted capture and sequencing spatial transcriptomics platforms, such as 10x Genomics Visium (Longo et al. 2021).

Our central objective in this study was to investigate the intratumor and intertumor heterogeneity in HGSC. We achieve this by spatially profiling three tissue sections and integrating with publicly available HGSC spatial transcriptomic cohorts. We assess the spatial organization of biological pathways and key biomarkers within and across sections, evaluate spatially coordinated pathway activity and cell-type distributions, and perform spatially informed cell–cell communication analysis to identify ligand–receptor interactions enriched within distinct tumor regions and molecular subtype contexts.

## Results

### Experimental design and overview of profiling one HGSC tumor sample

With a single fresh-frozen HGSC tumor, we used the 10x Genomics Visium spatial gene expression platform to spatially profile *N* = 4 tissue sections from the same tumor to investigate the intratumor spatial heterogeneity (capture areas 1A, 2B, 3C, 4D). After applying quality-control (QC) metrics, the 1A capture area was removed from downstream analyses because there were fewer total UMI counts and detected genes per spot compared with other capture areas (Supplemental Fig. S1; Supplemental Table S1). In addition to the spatial profiling, we also leveraged a previously published paired scRNA-seq from the same tumor (16030X2) that was generated using the 10x Genomics Chromium platform (Hippen et al. 2021). After QC, the scRNA-seq data set had 3469 cells for downstream analysis. Because this was a small number of cells, we integrated the paired scRNA-seq data with scRNA-seq data from an additional *N* = 6 HGSC tumors (Supplemental Table S1) to ensure robust identification of cell types. All sequencing was performed at the Huntsman Cancer Institute (HCI) at the University of Utah. After this point, we refer to these samples as the Utah samples.

Five additional scRNA-seq samples published by Denisenko et al. (2024) were retrieved from the NCBI Gene Expression Omnibus (GEO; https://www.ncbi.nlm.nih.gov/geo/; accession number GSE211956) and were subsequently integrated with the scRNA-seq samples generated by HCI to yield a data set for use as a cell-type reference for deconvolution (Fig. 1).

**Figure 1.**
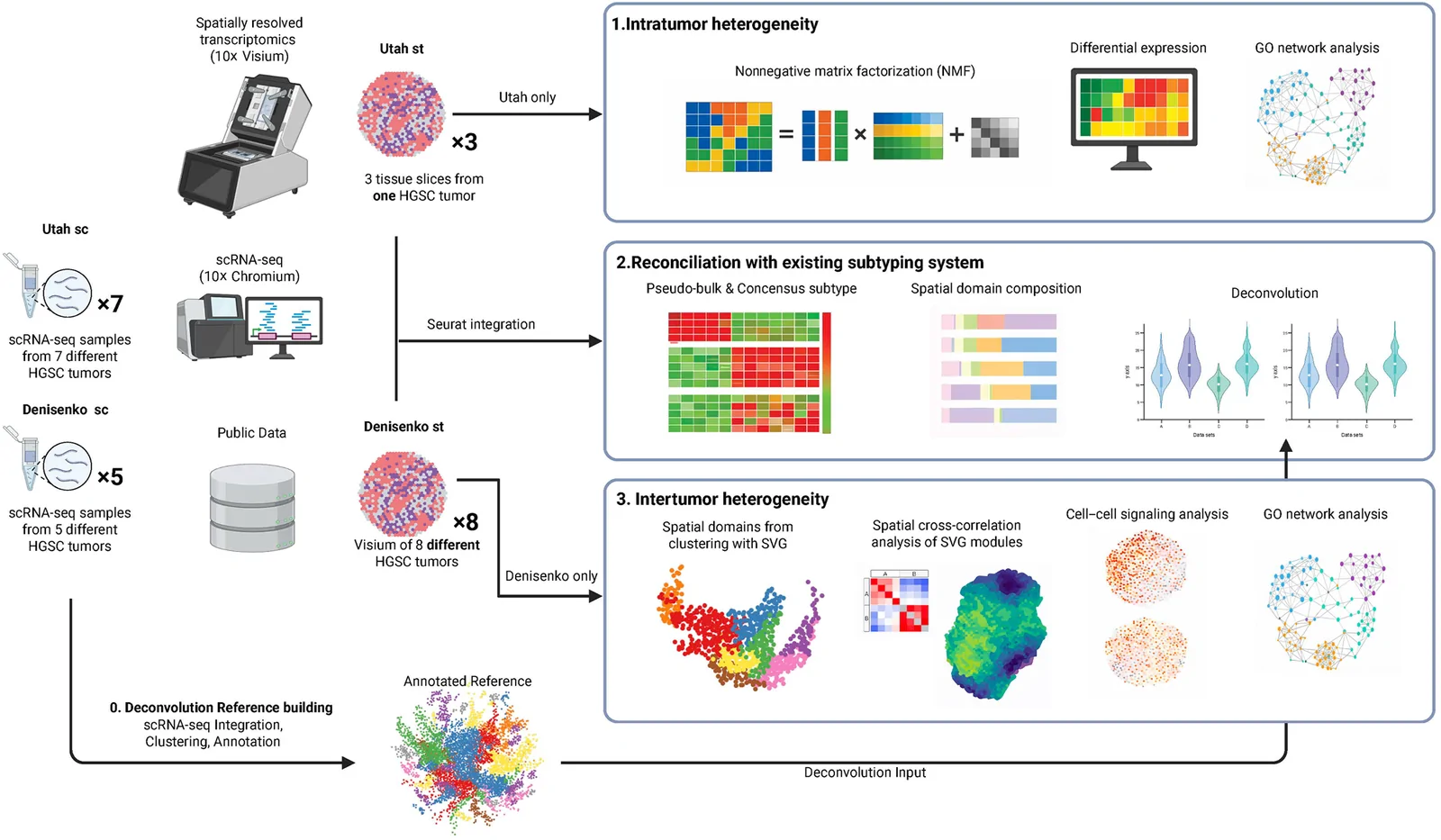
Integrated scRNA-seq and Visium spatial transcriptomics workflow for characterizing heterogeneity in high-grade serous carcinoma (HGSC). Public scRNA-seq and Visium data sets from HGSC tumors reported by Denisenko et al. (2024) are integrated with newly generated scRNA-seq and Visium data to investigate intra- and intertumor heterogeneity. All scRNA-seq data sets are jointly integrated to define cell states and to serve as a reference for downstream spatial transcriptomics deconvolution. Nonnegative matrix factorization (NMF) is applied to spatially variable genes in newly collected Visium samples from a single tumor to identify intratumoral spatial domains. All Visium samples are pseudobulked, consensus-subtyped, and deconvolved to assess the relationship between cell-type composition and established HGSC subtypes. Visium samples from Denisenko et al. are further analyzed to identify spatial domains using spatially variable genes (SVGs) and to perform Gene Ontology (GO) enrichment. Finally, spatial cross-correlation and cell–cell signaling analyses are conducted on a per-tumor basis to characterize intertumor heterogeneity and conserved spatial patterns.

### Characterizing intratumor heterogeneity using spatial transcriptomics

Our first objective was to characterize the spatial gene expression landscape across tissue slices derived from the same tumor tissue and to assess intratumor heterogeneity (Figs. 1, 2). To assess the heterogeneity at the spot level, we used a variable Bayes nonnegative matrix factorization (NMF) algorithm from the R/Bioconductor package ccfindR (Woo et al. 2019) to classify spots into *k* = 10 spatial domains. Despite deriving from the same tumor specimen, the three capture areas exhibited distinct spatial domain compositions and spatially variable gene expression. Spatial domain wise, capture areas 3C and 4D shared large contiguous regions dominated by NMF clusters 6 and 10. In contrast, capture area 2B lacked domains 8 and 10 and instead was characterized by an expanded cluster 3 domain enriched for immune-associated genes, indicating a localized immune-dominant niche distinct from the other regions (Fig. 2A–C). Slices 3C and 4D also shared similar spatial autocorrelation structures for gene expression, and 2B displayed a distinct immune-enriched signal, highlighting substantial intratumoral heterogeneity across adjacent tissue sections (Fig. 2D; Supplemental Fig. S2).

**Figure 2.**
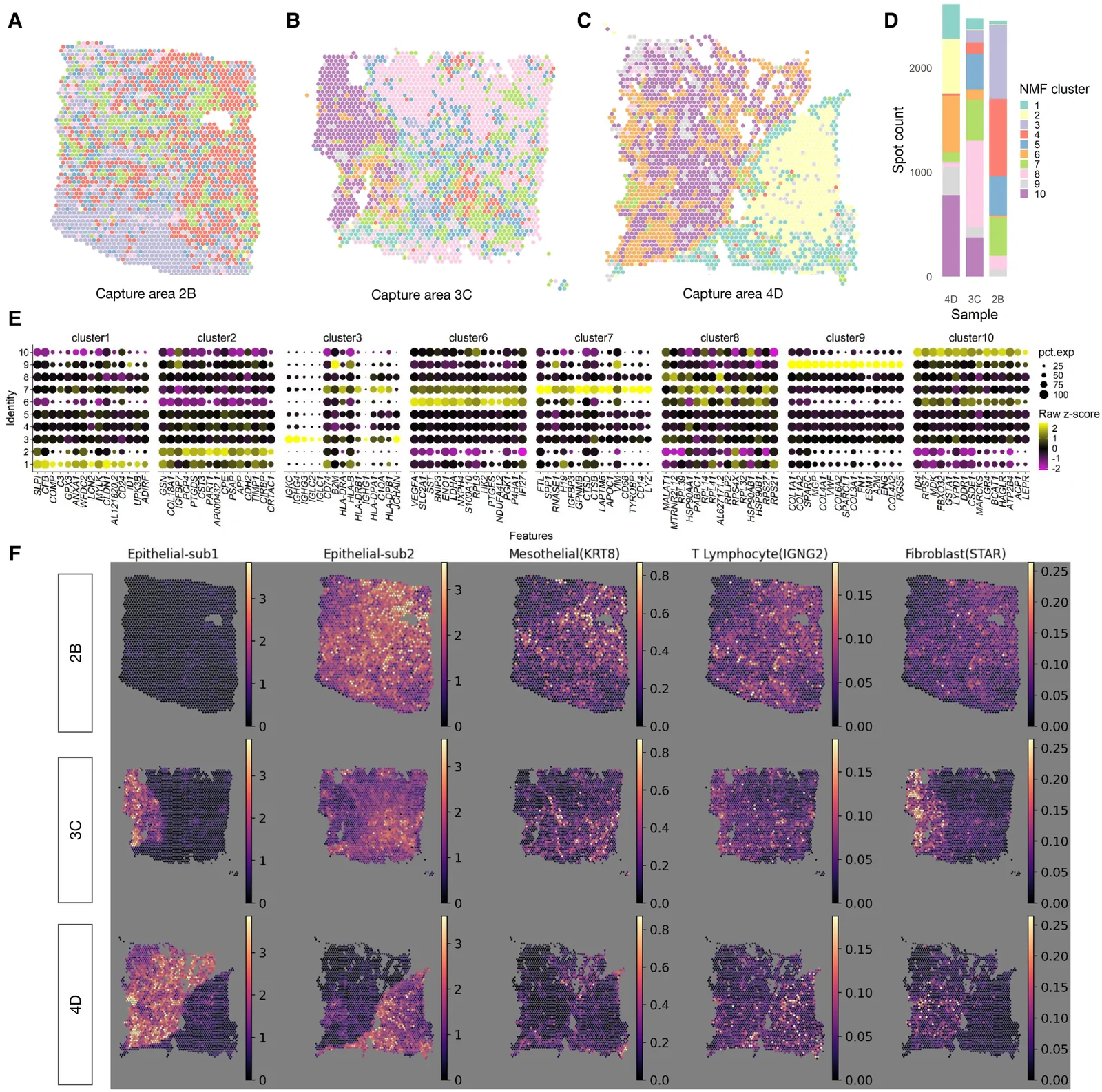
Characterizing intratumor heterogeneity using spatial transcriptomics. (*A*–*C*) Spot plot of predicted spatial domains (colors) using the R/Bioconductor package ccfindR (Woo et al. 2019) for *N* = 3 tissue sections (capture areas 2B, 3C, 4D) from a single HGSC tumor. (*D*) Stacked bar plot summarizing the proportions of predicted spatial domains across the three tissue sections. (*E*) Dot plot displaying expression (each scaled by standard deviation and centered at zero) of differentially expressed genes (DEGs) for the predicted spatial domains using Seurat (Hao et al. 2024) with default parameters. NMF clusters 4 and 5 had fewer than five significant DEGs and hence were omitted. (*F*) Visualization of cell2location (Kleshchevnikov et al. 2022) prediction of the fifth percentile of selected cell-type abundance for three capture areas (rows).

To characterize the molecular programs underlying these spatial domains, we identified differentially expressed genes (DEGs) for each NMF cluster (Fig. 2E). Cluster 1 was marked by established ovarian cancer biomarkers, including *WFDC2* and *SLPI* (Bingle et al. 2002; Tsukishiro et al. 2005). Cluster 3, composed predominantly of spots from 2B, showed enrichment for B cell and plasmablast-associated genes such as *IGKC*, *IGHG3*, *IGHG4*, and *JCHAIN*, consistent with an immune-dominant niche. Cluster 6 exhibited upregulation of *VEGFA*, known to be associated with endothelial activity and vasculature development, and *S100A10*, a hallmark gene for cancer migration (Noye et al. 2018), alongside glycolytic genes *LDHA* and *ENO1*, reflecting angiogenic and metabolic tumor programs (Feng et al. 2018; Huang et al. 2022). Cluster 7 was characterized by *SPP1*, known to be expressed by mesothelial cells and tumor-associated macrophages (Matsubara et al. 2023).

To orthogonally validate the domain structure identified by NMF, we applied cell2location (Kleshchevnikov et al. 2022) to infer cell-type abundance using a matched subset of scRNA-seq reference (Supplemental Figs. S3–S5). The putative malignant epithelial subcluster 1 population was mapped almost exclusively to the spatial domains of capture area 3C and 4D, whereas the epithelial subcluster 2 population mapped to all capture areas (most abundantly to 2B), recapitulating the spatial domain architecture observed in samples 3C and 4D yet absent in 2B, building additional evidence for intratumoral heterogeneity across adjacent capture areas, and suggesting the need for collecting multiple capture areas from the same tumor for a more comprehensive representation (Fig. 2F; Supplemental Fig. S3A–C; Forjaz et al. 2025). Mesothelial cells abundance estimation is consistent with NMF cluster 7 and its molecular program, whereas T lymphocyte and fibroblast abundance corresponds less strongly to NMF discovered spatial domains. It is worth noting that the matched single-cell reference sample 16030X2 contained predominantly putative malignant epithelial subclusters, with comparatively fewer stromal and immune populations relative to both itself and the full integrated reference (Supplemental Figs. S4C, S5D,E).

We further characterized the functional aspect of the NMF spatial domains using GO enrichment analysis on the domain-specific DEGs and visualized the enrichment network (Supplemental Fig. S6). Spatial domains predominantly observed in capture areas 3C and 4D (NMF clusters 9 and 10) were enriched for epithelial morphogenesis and extracellular matrix remodeling, suggesting differentiation and stromal organization. In contrast, domains largely restricted to 2B (NMF clusters 3 and 4) were enriched for immune-related functions including antigen presentation, B cell–associated immune activation, and leukocyte-mediated immunity. Spatial domains that are shared across the tissue slices reflect more general tumor-associated programs. Overall, the enrichment analysis seemed to suggest a model in which spatial differences in immune infiltration are linked to the segregation of domains across slices 2B, 3C, and 4D.

Taken as a whole, the convergence of findings in spatial domain structure, cell-type deconvolution, and functional enrichment suggest existence of substantial intratumoral heterogeneity between adjacent capture areas of the same HGSC tumor.

### Evaluating consensus HGSC tumor subtypes using single-cell and spatial transcriptomics samples

In this section, we examine the intra- and intertumor spatial heterogeneity in the context of established HGSC consensus subtypes. By leveraging scRNA-seq and spatially resolved transcriptomic data sets, we sought to understand if and how tumor composition varies within and between tumors and how that is associated to the canonical HGSC subtypes.

To support this analysis, we expanded beyond the initial *N* = 3 Utah HGSC Visium samples (all derived from a single tumor) and integrated them with a public Visium data set (Denisenko et al. 2024), comprising *N* = 8 HGSC tumors (one capture area per tumor; samples SP1–SP8). We aggregated molecular signals across all spots within each Visium capture area to generate pseudobulk expression profiles. This aggregation was used to assess how spatial samples align with existing HGSC subtyping frameworks in terms of cell-type composition and whether the *N* = 3 capture areas derived from the same tumor would receive consistent classification. For this, we used the cOV (Chen et al. 2018) R/Bioconductor package to classify the HGSC tumors into *k* = 4 subtypes. Within this package, we considered four established bulk-based HGSC classifiers: (1) the cOV method, (2) the Konecny method, (3) the Verhaak method, and (4) the Helland method (Helland et al. 2011; Verhaak et al. 2012; Konecny et al. 2014; Chen et al. 2018). This resulted in a total of *N* = 11 Visium capture areas spanning *k* = 4 established HGSC consensus subtypes defined by TCGA: differentiated (DIF), immunoreactive (IMR), PRO, and MES (Fig. 3A,B).

**Figure 3.**
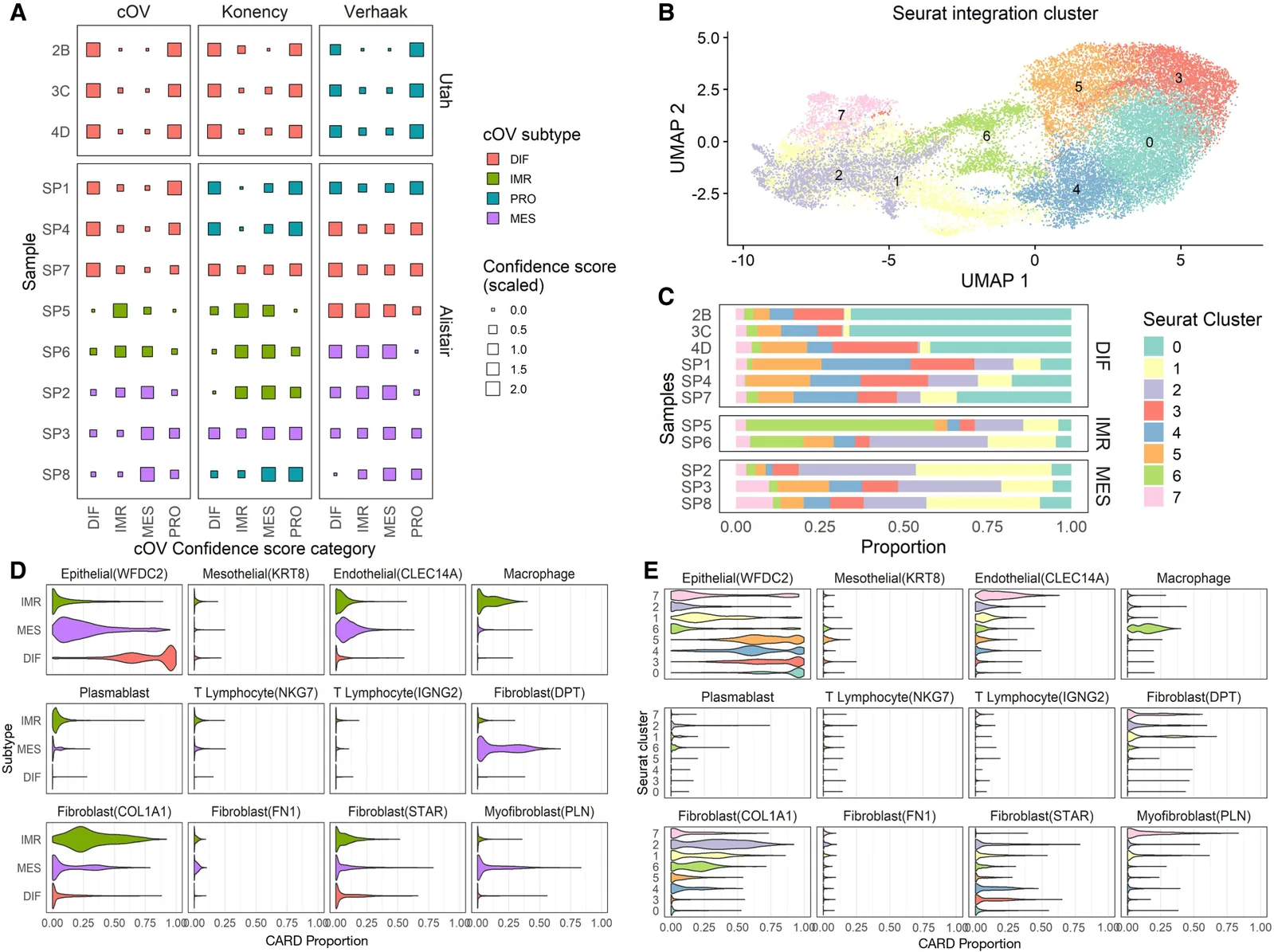
Characterizing intertumor heterogeneity using spatial transcriptomics. (*A*) Comparison of subtype assignment from four subtype prediction algorithms implemented by the consensusOV (cOV) R/Bioconductor package using pseudobulk RNA-seq profiles of *N* = 3 Utah HCI Visium samples and *N* = 8 Visium samples from Denisenko et al. (2024). (*B*) UMAP visualization of an integrated HGSC data set (from *N* = 11 HGSC capture areas). Colors represent the Seurat cluster of the integrated data set. (*C*) Bar plot showing the spot-level predicted spatial domains for all *N* = 11 Visium samples, with samples grouped by their predicted cOV HGSC subtype. (*D*) Violin plot showing the distribution of estimated cell types after applying spot-level deconvolution using CARD (Ma and Zhou 2022) for select cell types in the integrated data set, aggregated over differentiated (DIF), mesenchymal (MES), and immunoreactive (IMR) pseudobulk cOV HGSC subtypes. (*E*) Violin plot showing distribution of estimated cell-type proportions using CARD across the eight predicted spatial domains.

Across the four classifiers, we observed varying degrees of concordance in how pseudobulk Visium profiles aligned with consensus HGSC subtypes. The cOV and Konecny methods showed broad agreement in eight of the 11 capture areas, whereas the Verhaak and Helland methods exhibited greater divergence, including discrepancies in both subtype alignment and confidence scores across multiple samples (Fig. 3A).

Using the same pseudobulk-aligned samples, we next examined whether spatial domain structure exhibited commonalities within or across consensus subtype groupings. To this end, we visualized spot-level spatial domains alongside subtype reference labels to explore intra- and intertumor spatial variation (Fig. 3B). We observed substantial heterogeneity in the spatial distribution of domains both within tumors sharing similar subtype alignment and across different subtype groupings, underscoring the complexity of spatial organization beyond bulk-derived subtype categories (Fig. 3B,C; Supplemental Table S2; Chen et al. 2018).

We then performed spatially aware spot-level deconvolution for each of the *N* = 11 capture areas using the conditional autoregressive deconvolution (CARD) method (Ma and Zhou 2022) to estimate the relative cell-type proportions per spot. For visualization and comparison, samples were grouped according to their cOV-aligned subtype reference labels. The resulting cell-type composition patterns broadly resembled trends reported in prior bulk and spatial studies, providing contextual support for the interpretability of these subtype frameworks in aggregated spatial data. For example, capture areas aligned with DIF-associated profiles showed enrichment for epithelial cells and low fibroblast and macrophage proportions, whereas MES-aligned samples showed increased fibroblast and plasmablast proportions, and IMR-aligned samples exhibited elevated macrophage proportions (Fig. 3D; Hippen et al. 2023).

Finally, we examined CARD-estimated cell-type proportions stratified by spatial domains identified using Seurat (Fig. 3E). This analysis was performed to explore whether specific spatial domains tend to be associated with recurring cellular compositions across samples. We observed that domains more prevalent in DIF-aligned samples (0, 3, 4, 5) were enriched for epithelial cells with minimal fibroblast and macrophage presence, whereas domains more common in MES-aligned samples (1, 2, 7) were characterized by elevated fibroblast proportions and reduced epithelial content. Domain 6, predominant in IMR-aligned samples, exhibited increased macrophage proportions.

These patterns suggest recurring relationships between spatial domain structure and inferred cellular composition across tumors, while also highlighting variability within and between consensus subtype groupings. Together, these observations point to potential links between spatial organization and established HGSC subtype frameworks that will require further validation using patient-matched bulk, single-cell, and spatial measurements. Throughout the remainder of the study, the subtype labels derived here will be referenced to facilitate cross-sample comparisons. It is important to note that these labels function solely as a descriptive scaffold for presentation and interpretation and do not influence any subsequent analyses.

### Functional characterization of spatial domains in HGSC tumors

Next, we investigated biological functions underlying each of the predicted spatial domains within the HGSC TME. We sought to define the biological programs underlying each spatial domain identified within HGSC tumors. To systematically characterize these domains, we adopted a two-step strategy analogous to our Utah intratumor analysis. First, we integrated the Denisenko Visium samples (Denisenko et al. 2024) and performed unsupervised clustering, DE analysis, and annotation to define the spatial transcriptional landscape. We then conducted Gene Ontology (GO) enrichment analysis to functionally interpret domain-specific gene programs. Because Visium spots capture mixtures of cell types, domain-associated transcriptional differences may partly reflect variation in cellular composition rather than tumor-intrinsic regulatory states. To address this, we performed an additional composition-aware filtering step, excluding composition-driven DEGs prior to enrichment analysis.

### Unsupervised integration reveals epithelial-, fibroblast-, and immune-dominant spatial domains consistent with deconvolution results

The unsupervised clustering analysis produced *k* = 12 spatial domains after integrating the Denisenko samples (Fig. 4A,B). We then annotated the spatial domains using canonical epithelial, MES and immune markers. Five spatial domains (0, 2, 7, 9, 10) had a high expression for a known marker for ovarian cancer *WFDC2*. Therefore, we considered these domains to represent the epithelial-dominant parts of the tumor (Hellström et al. 2003). Notably the epithelial-dominant domains mostly comprised of spots from the DIF samples. Five other spatial domains (3, 5, 6, 8, 11) were marked by upregulation of gene sets involved in extracellular matrix organization, extracellular structure organization, and collagen fibril organization. We annotated these domains as the fibroblast-dominant/stromal portion of the tumor. These spatial domains are also most abundant with spots from the MES and IMR samples. Domain 4 displayed DEG sets associated with cytokine-mediated signaling pathways, antigen processing and presentation, innate immune responses, and several macrophage markers (*LYZ*, *CD68*) and was potentially representative of a mixture of infiltrating immune cells and tumor-associated macrophages. Domain 1 had fewer DEGs and seemed to express moderate levels of both epithelial and stroma marker genes (Fig. 4C; Supplemental Fig. S7). Notably, spots in domain 1 were primarily localized between epithelial-dominant and fibroblast-dominant domains, further suggesting that it may represent a transitional area between the stroma and epithelial cells (Supplemental Fig. S8).

**Figure 4.**
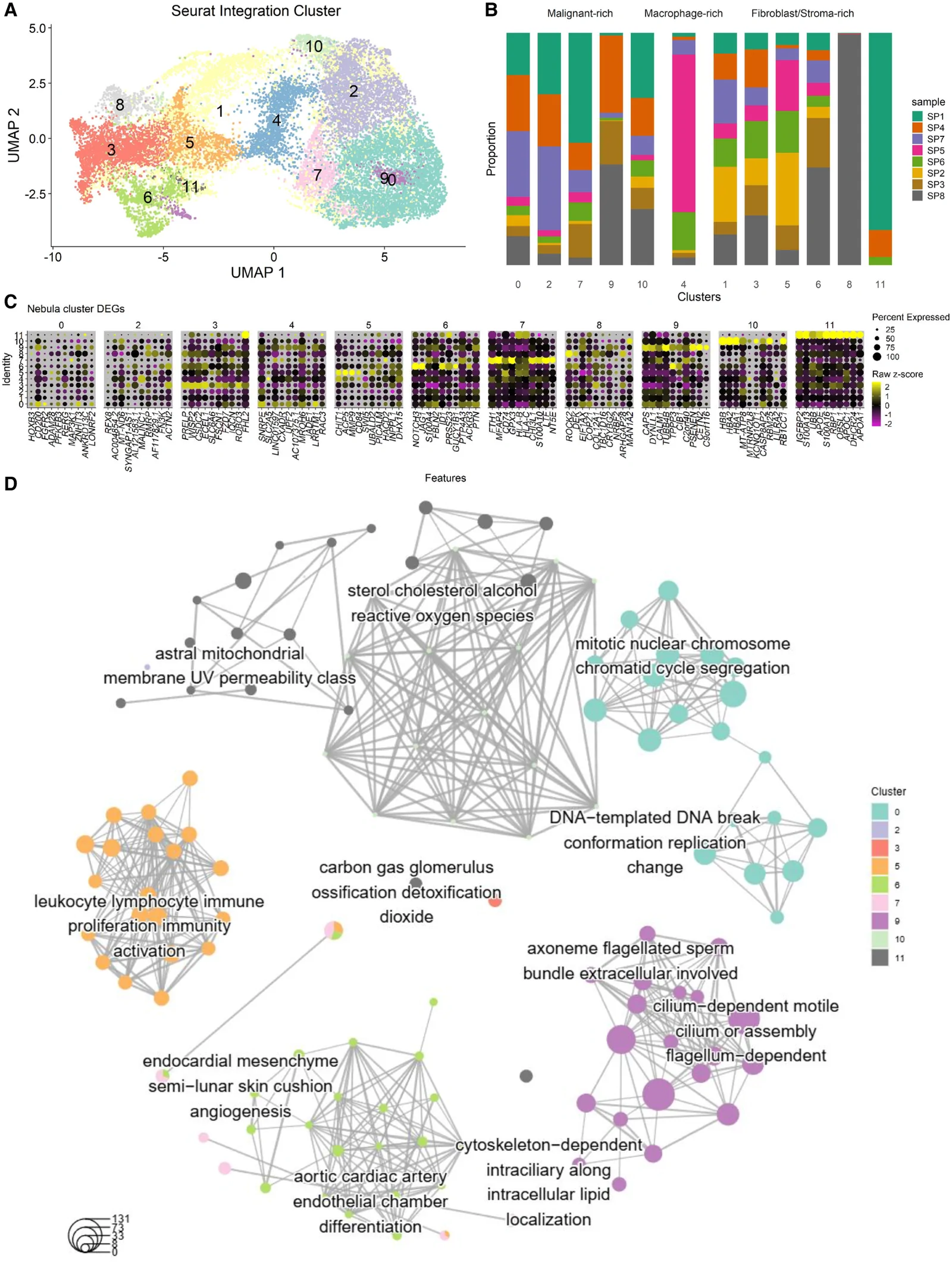
Functional characterization of predicted spatial domains in HGSC tumors. (*A*) UMAP representation of the *N* = 8 Denisenko (Denisenko et al. 2024) integrated Visium samples colored by *k* = 12 predicted spatial domains. (*B*) Stacked bar plot representing the relative proportions of spots sourced from each sample stratified by each predicted spatial domain. (*C*) Dot plot of marker gene expression of cluster-specific DEGs. Spatial domain 1 had fewer than five significant DEGs and hence was omitted. (*D*) Gene concept network plot showing the Gene Ontology (GO) results using clusterProfiler (Wu et al. 2021b) based on spatial domain-specific, upregulated DEGs sets with the edges indicating the biological pathways.

### Enrichment analysis on composition-corrected DEGs reveal domain intrinsic functional insights

Having established the architectural organization of the tumors, we next examined whether the transcriptional programs defining these domains persisted after accounting for cellular composition. Using CARD-estimated cell-type proportions as covariates, we performed differential expression analysis on spatially variable genes from the integrated Visium samples to separate domain-specific DEGs from those positively associated with cellular composition.

As an analysis baseline, we first performed the enrichment analysis on the composition-associated DEGs. As expected, the results are dominated by highly specialized, lineage-dependent functions such as localized glycolysis, monoatomic ion transport, and extensive leukocyte activation. This recapitulates the known biology of the cellular neighborhood (Supplemental Fig. S9; Gaudette et al. 2020; Galenkamp and Commisso 2021).

We then focused on the composition-corrected DEGs and applied functional enrichment to isolate tumor-intrinsic spatial programs. The corrected analysis yielded a network with more interconnected clusters. The epithelial-dominant domains are characterized by genomic instability, replication and repair (domain 0), cellular transport and motility (domain 9), and nonglycolytic metabolism (domain 10), all of which functions are unseen in the composition driven baseline network. We also observe varied functions in domain 7 spanning differentiation, adhesion, motility, and metabolism (Fig. 4D; Supplemental Fig. S10).

Likewise, the stromal domains 3 and 6 are characterized by unique structural differentiation functions pertinent to epithelial-to-mesenchymal transition, consistent with the observed colocalization of these domains with the epithelial-dominant domain 0 (Fig. 4D; Supplemental Figs. S8, S10).

Together, these analyses demonstrate that HGSC tumors are organized into spatially coherent epithelial, stromal, and immune niches, not explainable by cell-type enrichment alone. Furthermore, the bulk of the revealed functional diversity seemed to take place in the epithelial domains across tumors.

### IMR HGSC subtype areas have anticorrelation between immunoglobulin and major histocompatibility complex genes

Given the prominent immune infiltration observed in several spatial domains, we next examined whether immune-related gene programs were spatially coordinated within tumors and whether such coordination differed across HGSC molecular subtypes. We selected the top DEGs from the macrophage dominant cluster, plus two isolated immune gene programs (histocompatibility and immunoglobulin DEGs) and calculated the correlation between their expression (Fig. 5).

**Figure 5.**
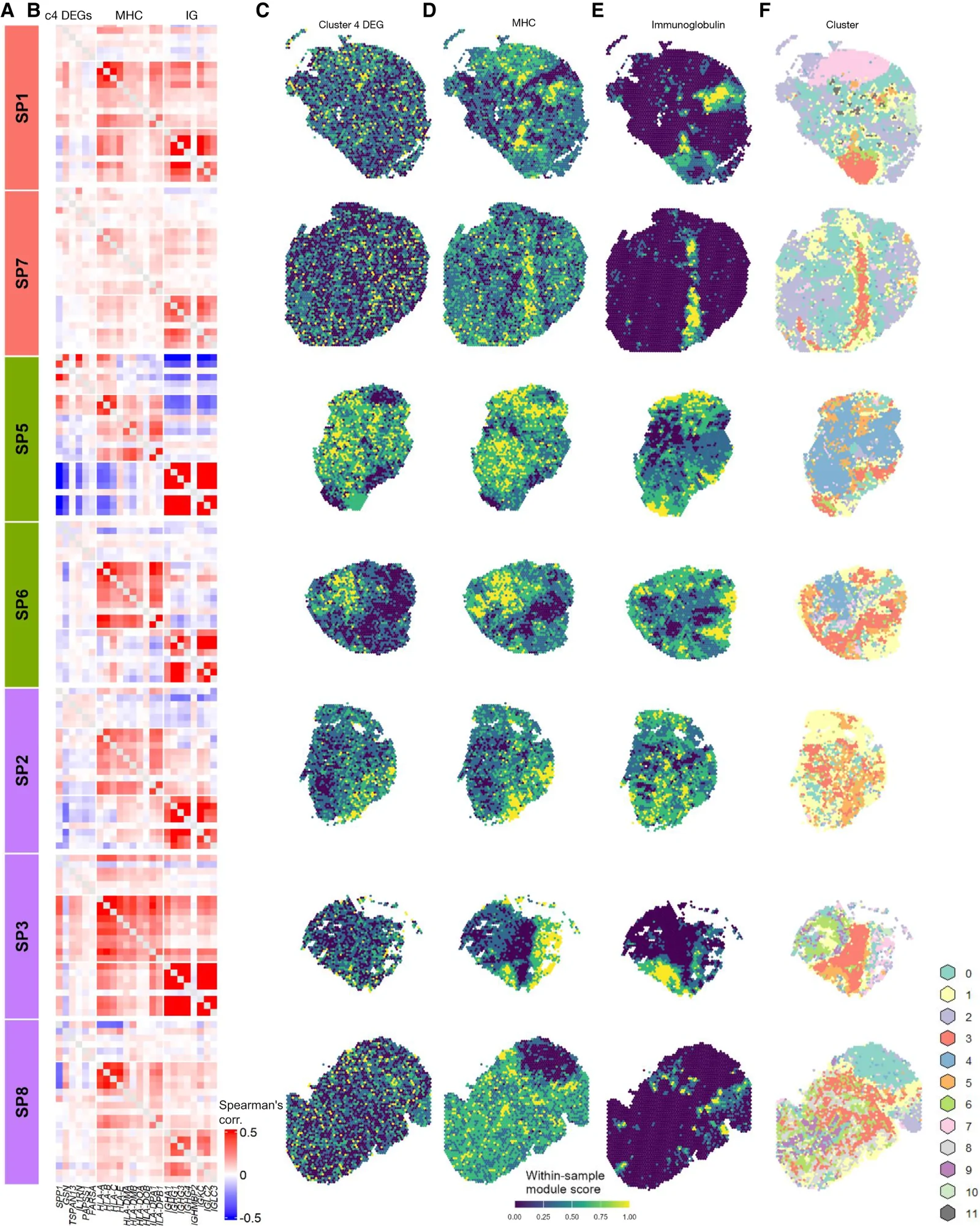
Immunoglobulin and major histocompatibility complex genes are anticorrelated in the immunoreactive (IMR) subtype, but not in the differentiated (DIF) or mesenchymal (MES) subtypes. Data visualization of predicted spatial domains using Seurat in select Denisenko Visium samples. We do not include the spatial domain visualization for sample SP4 (DIF) here, as it displays much less variability in spatial domains in comparison to other DIF samples. For SP4 spatial domains, please see Supplemental Figure S11. (*A*) Sample ID and color-coded molecular subtype: DIF in orange, IMR in green, and MES in purple). (*B*) Spearman's correlation values between domain 4 DEGs, major histocompatibility complex (MHC) genes and immunoglobulin (IG) genes. There was a negative correlation between MHC and IG exclusively for the IMR samples. (*C*) Column corresponding to the Seurat module score of DEGs identified from domain 4. (*D*) Column corresponding to the Seurat module score (average gene program expression) of major histocompatibility complex coding genes. (*E*) Column corresponding to the Seurat module score of immunoglobulin coding genes. Note that spots tend to coexpress high MHC and IG in the DIF and MES samples (*top* and *bottom* two rows), whereas spots expressing high MHC tend to anticolocalize with spots expressing high IG in IMR samples (rows color-coded green). (*F*) Integrated clustering scheme in space for reference.

Across the eight tumors, correlation structures varied by molecular subtype. Most notably, IMR tumors exhibited a consistent negative correlation between type II MHC genes and immunoglobulin genes (Fig. 5A,B; Denisenko et al. 2024). In the samples with a DIF subtype or MES subtype, we found zero to weak positive Spearman's correlations between these two gene sets. The spatial variability in these gene sets that is observed in other DIF subtype samples is not apparent in SP4 (DIF) (Supplemental Fig. S11). Expression of these MHC and immunoglobulin coding genes, if both were expressed, also colocalized in space (Fig. 5C–F). In samples with the IMR subtype, we found a negative Spearman's correlation between type II MHC and immunoglobulin coding genes (Fig. 5B), and expression patterns of the two immune gene sets seem to anticolocalize in space (Fig. 5C–F). The separation of immunoglobulin expression from histocompatibility antigen expression, which seemed to exclusively occur in the IMR subtype samples, may indicate plasmablast differentiation and movement away from the site of antigen presentation.

To validate whether the observed subtype-specific IG–MHC correlations are biologically significant as opposed to arising from random spatial structure or underlying cellular composition, we performed two complementary validation analyses. First, we generated empirical null distributions by permuting spot-level module scores and gene expression values, allowing us to estimate the probability of observing correlations of similar magnitude by chance. Second, we computed partial correlations while controlling for deconvolution-estimated cell-type fractions, with and without additional adjustment for spatial coordinates, to assess whether compositional heterogeneity or spatial autocorrelation explained the observed associations. Although composition correction attenuated several associations, most notably the IG–MHC anticorrelation in IMR samples, not all signals were eliminated, indicating that aspects of the spatial organization persist beyond compositional heterogeneity (Supplemental Fig. S12A–E).

### Investigating cell–cell communication in HGSC tumors across spatial domains

Lastly, we applied COMMunication analysis by Optimal Transport (COMMOT) (Cang et al. 2023) to infer spatial ligand–receptor communication across tumors. A total of 172 ligand-receptor pairs from the CellChatDB database (Jin et al. 2021) were found to be expressed in at least five spots across all Denisenko samples. Several signaling programs, including MK, MIF, chemokine, and complement pathways, were recurrently detected. Communication strength distributions were largely consistent within molecular subtypes, with IMR and MES tumors displaying higher mean signaling levels compared with DIF tumors. In addition to differences in overall signaling magnitude, subtype-specific spatial correlation patterns among ligand–receptor pairs suggested distinct organizational architectures of signaling activity across tumors (Supplemental Fig. S13A,B).

We then examined the correlation between ligand-receptor pairs and found subtype-specific correlation patterns. Most notably, the high within-pathway correlation of the MK pathway in sample SP5 suggests the lack of differential colocalization of *MDK* mediated signaling in space. In contrast, in samples SP1 and SP7, in which *MDK*-*LRP1*, *MDK*-*NCL*, and midkine-syndecan 4 (*MDK*-*SDC4*) signaling colocalize to different clusters, there is little intra-MK correlation. The within-pathway correlation of the MIF pathway is relatively high for IMR samples SP5 and SP6 as well as MES sample SP2 and is much less in the DIF samples SP1, SP4, and SP7 (Supplemental Fig. S14).

### Ligand-receptor signaling occurs in different spatial domains across HGSC samples pseudobulk-aligned as DIF

midkine-syndecan 1 (*MDK*-*SDC1*) signaling is virtually globally active in SP1, although only weakly present in specific clusters (5, 7) of SP4 and SP7. The *MDK*-*SDC4* signaling is active in SP4 and SP7 in a global manner, although only prominent in cluster 7 of SP1 (Fig. 6A,B). This observed differential regulation of syndecan 1 and 4 across the pseudobulk-aligned DIF subtype samples may indicate distinct functions underlying these ligand-receptor interactions and is potentially consistent with the results from a previous study on breast carcinoma, which reported a significant association between syndecan 1-positive staining and syndecan 4-negative staining with tumor grade, suggesting they are independent indicators in breast cancer (Lendorf et al. 2011).

**Figure 6.**
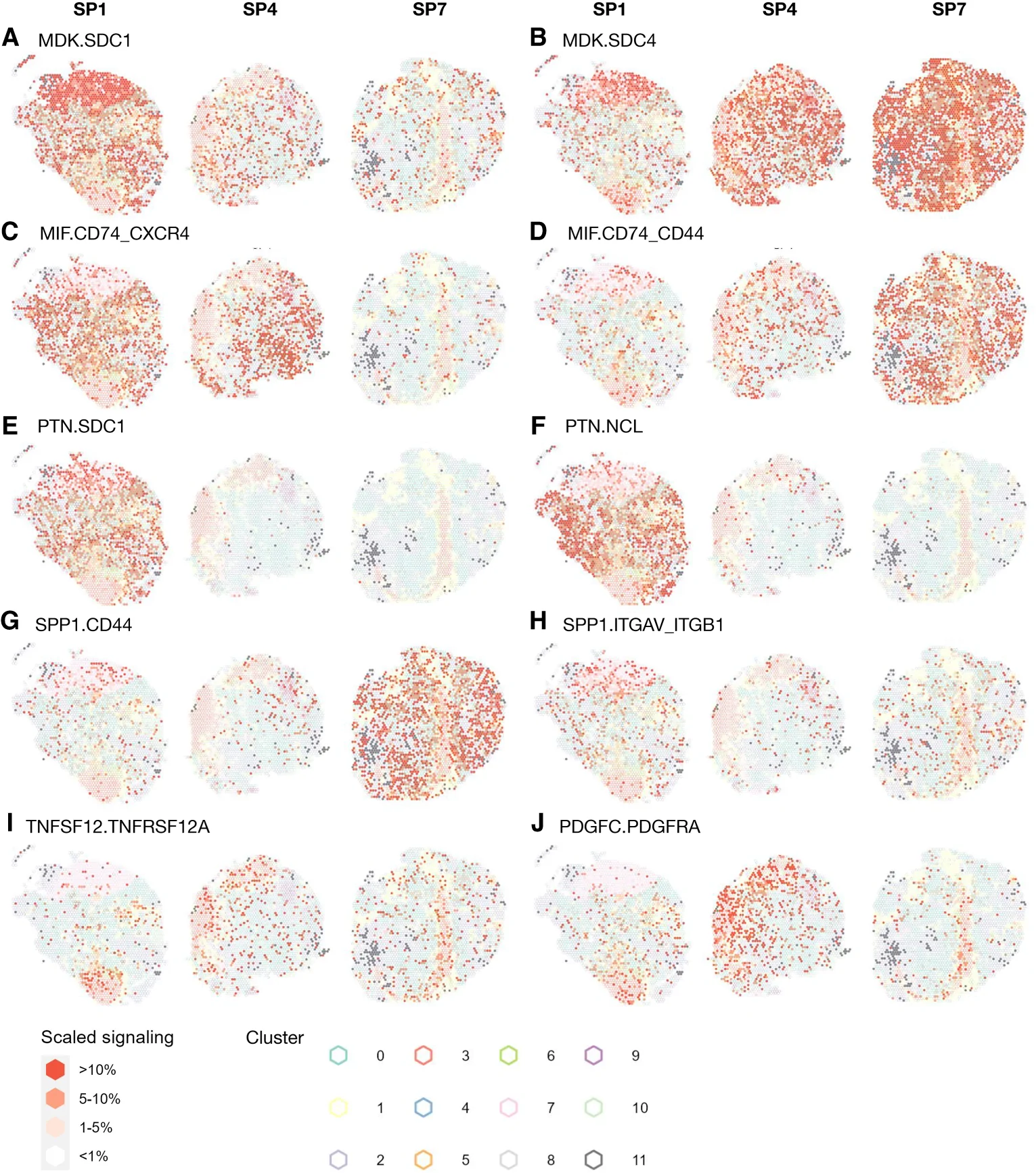
Ligand-receptor signaling occurring in different domains across samples of the DIF subtype. Each panel (*A*–*J*) displays the outgoing signal strength predicted by COMMOT for a different ligand-receptor pair in three DIF samples SP1, SP4, and SP7, respectively. The fill of the spots represents binned signaling across all spots; the *outer* boundary is color-coded by the spatial domain.

*MIF*-*CD74*-*CXCR4*, the noncognate ligand receptor interaction that is reported to have chemokine-like functions and result in recruitment of immune cells, has prominent signaling present in the epithelial cell–rich clusters of SP1 and SP4 (clusters 0, 9, 10, 11) although was much lower in SP7. *MIF*-*CD74*-*CD44*, the canonical interaction that triggers the standard ERK MAP kinase pathway activation, is substantially upregulated in SP7 and only present in clusters 5, 6, and 7 in SP1 and SP4 (Fig. 6C,D; Noe and Mitchell 2020). A recent study featured reported cell–cell communication between macrophages and T cells in ovarian cancer via the *MIF*-*CD74*-*CXCR4* pathway altering CD8-T cell function and impacting prognosis (Wang et al. 2022). This difference in MIF signaling observed within the pseudobulk-aligned DIF subtype is potentially reflective of the differing immune cell state between SP7 and the other samples aligned to DIF, especially under the observation that SP7 had a substantially higher proportion of spots of the immune cell–dominant cluster 4 (Fig. 4B).

*PTN* is known as a mitogenic cytokine whose signaling is associated with neural development as well as various cancer-related biological activities (Wang 2020). PTN signaling appears exclusively in the epithelial cell–dominant clusters (0, 7, 10) of sample SP1 and no other sample (Fig. 6E,F).

*SPP1*-*CD44*, a pathway known to carry out immunosuppressive functions (Cheng et al. 2023), is globally active in SP7 and absent from the two other pseudobulk-aligned DIF samples. *SPP1*-*ITGAV*- *ITGB1* interaction, which is reported to promote tumor progression in ovarian cancer (Zeng et al. 2018), is present in all three pseudobulk-aligned DIF samples in moderate levels and yet appears to colocalize with different clusters. In SP1, *SPP1*-*ITGAV*-*ITGB1* signaling happens primarily along the boundary lines of cluster 7 as well as along the division of clusters 0 and 1; In SP4, *SPP1*-*ITGAV*-*ITGB1* colocalizes almost exclusively with cluster 9; in SP7, *SPP1*-*ITGAV*-*ITGB1* is most prominent along the fibroblast-dominant clusters 3, 5, and 6 and cluster 7 (Fig. 6G,H; Supplemental Fig. S15; Supplemental Table S3).

The TWEAK-Fn14 (*TNFSF12*-*TNFRSF12A*) interaction, known to activate cellular processes such as proliferation, invasion, and angiogenesis in cancer (Winkles 2008), is present in all three pseudobulk-aligned DIF samples in moderate levels and colocalizes primarily with fibroblast rich cluster 3. In SP4 and SP7, this interaction is additionally observed in the epithelial cell–dominant clusters 0 and 7 (Fig. 6I,J).

Such differences in cell–cell communication pathways between pseudobulk-aligned DIF samples are indicative of the heterogeneity of tumors within the same molecular subtype. Differences in signaling patterns include both global differences in signaling strength and the specific colocalization of signaling with spatial domains, further highlighting the presence of unique tumor architectures.

### Ligand-receptor signaling is largely conserved intratumor despite transcriptional and spatial variability

To assess whether spatial communication heterogeneity is preserved within a single tumor, we applied the same pipeline to three Utah sections derived from one pseudobulk-aligned DIF tumor. In contrast to the marked intertumoral and intrasubtype variability observed in the Denisenko (Denisenko et al. 2024) cohort, the Utah sections demonstrated highly concordant signaling landscapes, with reproducible pathway activity and preserved intrapathway correlation structure (Supplemental Fig. S16).

Notably, despite detectable regional differences in spatial gene expression within the Utah specimen, inferred cell–cell communication architectures remained comparatively stable, indicating that higher-order signaling networks may be more conserved compared with localized transcriptional programs (Fig. 2; Supplemental Fig. S16).

Collectively, these analyses demonstrate that spatial ligand–receptor communication in HGSC is shaped by both molecular subtype and tumor-specific architecture. Across the Denisenko (Denisenko et al. 2024) cohort, we observed marked intertumoral and intra-DIF-subtype variability in signaling magnitude, pathway coordination, and spatial colocalization patterns. In contrast, replicate sections derived from a single Utah tumor exhibited highly concordant communication landscapes, with preserved pathway activity and intrapathway correlation structure despite regional transcriptional heterogeneity. These findings suggest that although higher-order signaling architectures are reproducible within individual tumors, they diverge meaningfully across tumors and within subtypes, underscoring the presence of intertumor heterogeneity in HGSC beyond what existing bulk classification systems can measure.

## Discussion

Utilizing spatial transcriptomic data from several sections from the same tumor, we explored intratumor heterogeneity. We also expanded this analysis to intra- and intertumor heterogeneity by integrating publicly available HGSC tumors.

Our findings on the variability of the Visium data set taken from the same tumor sample is potentially suggestive of the limited ability for a single Visium tissue slice to comprehensively characterize the complex TME and calls for the need to generate multiple Visium slices. However, a technical limitation underlying our Visium samples is that we sequenced fresh-frozen tissue, which may result in undesired tissue stretching and is more vulnerable to poor quality tissue from RNA degradation compared with formalin-fixed paraffin-embedded (FFPE). Replication of these findings using FFPE tissue will be needed to better validate our observed patterns of intratumor heterogeneity.

The application of spatially aware deconvolution, using the CARD package, with a molecular subtype classifier, cOV, on the pseudobulk of Visium samples allowed us to identify a connection between tumor composition and molecular subtype. Our analysis of spatially resolved transcriptomic data confirms the significance of molecular subtypes of HGSC despite the existence of considerable intertumor variability and complexity of solid tumor tissue. Limitation of this analysis lies in a single-cell reference annotation step, in which malignant epithelial identity is inferred exclusively from transcriptomic lineage markers, tumor-associated expression programs, and clustering structure rather than more definitive evidence such as CNV inference owing to limitations of the short-read sequencing data. Therefore, we conservatively refer to epithelial populations as putative malignant epithelial subclusters. Future work should incorporate paired genomic profiling or long-read sequencing in reference data generation to enable more definitive malignant versus nonmalignant annotation.

Subsequent functional enrichment analysis on the integrated Visium samples revealed spatial domains with distinct biological functions shared across tumor tissues. In addition, using spot-wise Spearman's correlation between expression of DEGs, we detected an IMR subtype-specific negative interaction between MHC genes and immunoglobulin genes, which appears to be a novel finding and may indicate potential interaction of plasma cells and antigen-presenting cells. One possible explanation of this phenomenon is IMR subtype-specific movement of B cells away from the site of antigen presentation, which may indicate stronger or later-stage immune infiltration. However, we only analyzed two IMR samples in this paper, which limits our ability to arrive at concrete conclusions. Further research incorporating more HGSC samples with reliable molecular subtype classification will be needed to validate whether these interactions are prevalent in and specific to IMR samples. Moreover, spatially resolved transcriptomic technology at single-cell resolution, such as Vizgen MERFISH and 10x Genomics Xenium and can be leveraged to interrogate the spatial organization of plasma cell and antigen-presenting cells and confirm whether an anticolocalization occurs in IMR samples. A related limitation is that immune-enriched spatial domains were not interrogated in depth for organized immune architectures such as tertiary lymphoid structures (TLSs). Although immunoglobulin-rich domains may be biologically relevant to TLS-like immune organization, 10x Visium does not provide single-cell resolution, and immunoglobulin-enriched spots may instead reflect diffuse immune infiltration or localized plasma cell abundance rather than structured TLS formation. Further research incorporating more HGSC samples with reliable molecular subtype classification will be needed to validate whether these MHC–immunoglobulin spatial relationships are prevalent in and specific to IMR samples. Higher-resolution spatial transcriptomic technologies, such as Vizgen MERFISH, 10x Genomics Xenium, or complementary multiplex immunostaining, could further resolve the spatial organization of plasma cells, B cells, antigen-presenting cells, and TLS-associated immune structures in IMR HGSC.

It is also worth raising that the molecular subtype information incorporated in this study is derived from bulk expression–based frameworks that were originally developed for microarray and bulk RNA-seq assays and are used here as a contextual scaffold rather than as definitive classifications for individual spatial samples. Given the limited number of Visium capture areas per subtype, particularly for the IMR group, associations between spatial expression patterns, signaling activities, and consensus subtypes should be interpreted as provisional and descriptive. The observed convergence and divergence of spatial programs across tumors highlight how bulk-derived subtype definitions can intersect with, but do not fully capture, the complexity of spatially resolved tumor ecosystems.

Accordingly, the subtype-associated spatial patterns and signaling relationships reported here should not be viewed as subtype-restricted features but rather as preliminary organizational principles that emerge when spatial transcriptomic data are interpreted within an existing HGSC subtyping framework. The presence of substantial intrasubtype variability underscores the likelihood that multiple spatial configurations and microenvironmental states can coexist within the same bulk-defined subtype. Larger spatial cohorts, patient-matched single-cell and spatial data sets, and orthogonal validation approaches will be required to assess the robustness, prevalence, and biological significance of these patterns.

Lastly, we interrogated the cell–cell communication patterns of all samples across all DIF subtype samples. Our application of COMMOT, a spatially aware method based on optimal transport for cell–cell communication analysis, has unveiled considerable heterogeneity in signaling activity across tumors of different molecular subtypes. The differential colocalization of signaling activities to distinct spatial domains within each tumor could be a driving factor for recruitment of cells and thus shaping the TME. We identified potential cell–cell communication features we could extract from spatially resolved data, such as the strength of correlation between immune gene sets and the presence/absence of signaling pathways, that may be valuable covariates for consideration when assessing prognosis. Potential future directions include further analysis of spatially resolved data, searching for marker gene expression strongly associated with such spatial features to enable a survival analysis population level using bulk-RNA-seq as input.

Despite analyzing the same ovarian HGSC data set published (Denisenko et al. 2024), our cell–cell communication results are hardly comparable owing to the drastically different methodology being used (the Denisenko study focused on the correlation between expression of select ligand and Giotto cell-type abundance scores in a patient-specific manner, whereas the COMMOT package takes into account the spatial information and potential heterodimeric interactions).

A limitation of this study is the small number of Visium capture areas analyzed per tumor and per consensus subtype, which constrains the resolution at which spatial variability can be systematically characterized. Although multiple capture areas from the same tumor provide insight into intratumor heterogeneity, a single or small number of capture areas per tumor may not fully represent the spatial complexity of the TME, particularly for large or highly heterogeneous tumors. Consequently, spatial patterns observed within individual capture areas may reflect localized tissue architecture rather than global tumor-wide organization.

As spatial transcriptomics technologies continue to scale, future studies incorporating denser sampling across multiple regions of the same tumor and across larger patient cohorts will be essential to disentangle capture area–specific effects from reproducible tumor-level or subtype-associated spatial programs. Such designs will enable more robust estimation of spatial heterogeneity, facilitate assessment of reproducibility across capture areas, and improve the generalizability of spatial features identified in this study.

A second limitation of the study is the lack of the incorporation of patient-level metadata on therapy and survival. Analysis of more spatially resolved transcriptomics data with inclusion of patient level metadata should be done to verify our current findings and explore additional features in spatial data associated with treatment and survival. Additionally, the cell–cell communication analysis is subject to the limitation of having a predefined communication distance threshold (dis_thr) parameter. We selected a relatively small value of 250 for all samples, which only allowed for the detection of short distance secreted signaling. In conclusion, our study introduces integrative analytical approaches for single-cell and spatially resolved transcriptomics that contextualize spatial gene expression patterns, functional programs, and cell–cell communication within established HGSC molecular subtyping frameworks. Although interpretation is constrained by the use of bulk-derived subtype annotations as a reference scaffold, limited numbers of spatial samples per subtype and per tumor, and limited patient-matched single-cell data, our analyses highlight spatially resolved features and organizational principles that may not be apparent from bulk profiling alone. These findings motivate future studies incorporating larger spatial cohorts, denser tumor sampling, patient-matched modalities, and orthogonal validation to assess the robustness and clinical relevance of spatial features in HGSC.

## Methods

### Denisenko et al. HGSC spatial transcriptomics and scRNA-seq data

The Cell Ranger/Space Ranger processed count matrices and corresponding metadata of the *N* = 8 spatial transcriptomics data and *N* = 5 (*N* = 3 patient-matched) scRNA-seq data were retrieved from GEO (accession number GSE211956) (Denisenko et al. 2024). For the spatial transcriptomics data, the authors made available the standard outputs from 10x Genomics Space Ranger and Loupe Browser (filtered matrix, scalefactor, and tissue positions files) for *N* = 8 Visium data sets, which were used to construct SeuratObject S4 (v4.1.3) (Denisenko et al. 2024). However, subsequent analysis was performed with Seurat version 5.0.1. We did not remove any spots based on standard QC metrics including the percentage of reads mapping to mitochondrial genes, total number of UMI counts per spot, or number of detected genes. This resulted in a total number of 15,193 genes × 20,123 spots. Raw counts were normalized using Seurat SCTransform(), regressing out the percentage of mitochondrial genes, percentage of ribosomal genes, and total UMI counts. For the scRNA-seq data, the authors made available the count matrices (in tab-delimited file format) for the five samples (Y2, Y3, Y5, MJ10, MJ11). Cells expressing fewer than 200 genes or >25% mitochondrial genes were removed. Genes expressed in fewer than three cells were also excluded. The dimensions of our data set pre/post are detailed in Supplemental Table S4.

### Generation of Utah HGSC tumor spatial transcriptomics data

One fresh-frozen tumor sample (JD24379) was shipped from the HCI at the University of Utah to the Cedars-Sinai Applied Genomics, Computation & Translational Core on dry ice. The tumor was embedded in an optimal cutting temperature (OCT) compound per the demonstrated protocol for tissue preparation (demonstrated protocol CG000240). For RNA quality assessment, 10 sections of 10 µm thickness were collected in a precooled microcentrifuge tube, transported on dry ice, and stored at −80°C per the demonstrated protocol. RNA extraction was performed using the Zymo Quick-RNA micro kit, and RNA integrity was evaluated on the 2100 Bioanalyzer. Methanol fixation, H&E staining, and imaging were performed per the demonstrated protocol (demonstrated protocol CG000160). Tissue optimization was performed following 10x Genomics Visium spatial gene expression for fresh-frozen tissue optimization protocol (user guide CG000238). Spatial gene expression libraries were generated following 10x Genomics Visium spatial gene expression for fresh frozen protocol (user guide CG000239). Libraries were sequenced on an Illumina NovaSeq 6000 instrument. The length of read 1 was 28 bp, i7 Index and i5 Index were 10 bp, and read 2 was 90 bp.

### Generation of Utah HGSC tumor scRNA-seq data

Samples were collected from seven patients with advanced stage (III or IV) ovarian HGSC by the HCI at the University of Utah. Five patients underwent primary debulking surgery prior to adjuvant chemotherapy, and two patients (corresponding to samples 19833X1 and 19833X2) received three cycles of neoadjuvant chemotherapy prior to surgery followed by adjuvant chemotherapy.

In preparation for single cell RNA-seq, fresh macrodissected tumor samples (19459X1, 19595X1, 19833X1, 19833X2) were cut into 2 mm chunks and dissociated using the human tumor dissociation kit from Miltenyi Biotec (130-095-929), according to the manufacturer's instructions, in C tubes (130-093-237) with RPMI (Thermo Fisher Scientific 11875135) + 10% fetal bovine serum (FBS; Thermo Fisher Scientific 26140079). Dissociation was done on gentleMACS octo dissociator from Miltenyi Biotec for 1 h at 37°C. The cellular suspension was filtered using a 70 micron cell strainer (Miltenyi Biotec 130-098-462). Filtered cells were washed twice using a PBS + 0.04% FBS solution. Washed cells were diluted to obtain a concentration of 600,000–1,400,000 live cells/mL, followed by cryopreservation in liquid nitrogen.

Thawed dissociated cell suspensions were partitioned into an emulsion of nanolitersized droplets using a 10x Genomics chromium controller and chromium next GEM chip G, and RNA sequencing libraries were constructed using the 10x Genomics Chromium Next GEM Single-Cell 3′ Reagent Kit v3.1. Purified cDNA libraries were qualified on an Agilent Technologies 2200 TapeStation using a D1000 ScreenTape assay (5067-5582). The molarity of adapter-modified molecules was determined by quantitative PCR using Kapa Biosystems Kapa library quantification kits (KK4824; 0796014001). Individual libraries were normalized to 5 nM, and equal volumes were pooled in preparation for Illumina sequence analysis. Sequencing libraries were sequenced on an Illumina NovaSeq 6000 instrument using the NovaSeq 6000 S4 v1.5 reagent kit (20028312; paired-end, 150 × 150 bp).

Experimental details for samples 16030X2, 16030X3, and 16030X4 were previously reported (Weber et al. 2021), and data are available through dbGaP (accession phs002262.v2.p1). Briefly, tumor chunks cryopreserved in liquid nitrogen were thawed and dissociated into single cells using the Miltenyi human tumor dissociation kit and the GentleMACS dissociator. Library preparation was performed using the 10x Genomics 3′ gene expression library prep v3. Libraries were sequenced on an Illumina NovaSeq 6000 instrument.

### Data preprocessing and QC

For all analysis steps described, we used Python (version 3.7), R (version 4.3.1) (R Core Team 2021), Seurat (version 5.0.1) (Satija et al. 2015), and Bioconductor (version 3.18) for analysis of genomics data. Space Ranger output was read into Python to create an AnnData object and read into Seurat and Bioconductor to create SeuratObjects and spatial-experiment objects (Hao et al. 2024; Righelli et al. 2022).

#### Utah Visium raw data processing and QC

For the tumor sample (JD24379), FASTQ and image data were preprocessed with the 10x Space Ranger pipeline version 3.0.1. Reads were aligned to reference genome GRCh38-2020-A. SeuratObject S4 (v4.1.3) were constructed for the four Visium capture areas of the tumor sample, with subsequent analysis performed with Seurat version 5.0.1.

One out of four Utah HCI Visium samples had considerably fewer read counts mapped to each spot (3000–5000) compared with the others and was hence excluded from downstream analysis. For the other Utah Visium samples, we did not remove any spots based on standard QC metrics including the percentage of reads mapping to mitochondrial genes. After applying these QC steps (removal of capture area 1A), the number of gene by the number of spots was 18,162 genes × 7537 spots.

Raw counts were normalized using Seurat SCTransform(), regressing out the percentage of mitochondrial genes, percentage of ribosomal genes, and total UMI counts.

#### Utah scRNA-seq raw data processing and QC

Following sequencing, we mapped reads to GRCh38-2020-A using Cell Ranger (version 7.0.0). After alignment, we perform QC and dropped only cells expressing fewer than 200 genes or >25% mitochondrial genes. Genes expressed in fewer than three cells were also excluded. The dimensions of our data set before/after cell-level QC are detailed in Supplemental Table S1.

### Data analysis

#### Feature selection, dimensionality reduction, and batch effect correction for Visium samples

Features (genes) were selected using the Seurat FindSpatiallyVariableFeatures function using default parameters and selection.method = “moransi” for all individual SRT samples; variable genes identified in at least two samples were selected as integration features. Following feature selection, principal component analysis (PCA) was performed with Seurat RunPCA with npcs = 30. To visualize the spots from the integrated data set, we generated uniform manifold approximation (UMAP) representation of the principal components.

To correct for batch effects, we integrated the spots across the Visium samples using SelectIntegrationFeatures function in Seurat with 3000 features, FindIntegrationAnchors under reference = NULL and reduction = “cca”, and IntegrateData() using the 30 principal component dimensions for anchor weighting procedure while explicitly setting features.to.integrate as all genes.

#### Unsupervised clustering to identify predicted spatial domains for Utah Visium samples

To predict spatial domains, we used NMF. Specifically, the variable Bayes NMF function vb_factorize from the ccfindR package was run on the merged raw count matrices of spatially variable genes from the *N* = 3 Visium Utah samples, over ranks ranging from eight to 16, with five replicates per rank (Woo et al. 2019). The algorithm identified an optimal rank of 10 and assigned spots from all three samples into 10 predicted spatial domains.

#### Unsupervised clustering to identify clusters in the integrated Visium data set

Following integration with Seurat IntegrateData() as described previously, we ran the Seurat FindNeighbors() using all 30 principal components and then the FindClusters() function at resolution 0.4 and default Louvain algorithm to obtain the clusters (the same clustering methodology was applied to both Utah, Alistair/Denisenko et al. SRT integration and Denisenko et al. SRT integration).

#### Differential gene expression for Utah Visium sample across NMF predicted spatial domains

Preliminary differential expression analysis was performed on the integrated data set using Seurat FindMarkers() function under log fold-change.threshold parameter of 0.25 and min.pct parameter of 0.1, comparing spots from each NMF cluster (with more than 50 spots) with all other spots (in a one vs. all manner).

NEBULA was used for the formal differential expression analysis. The integrated SeuratObject of the three Utah samples was supplied to the NEBULA scToNeb() function, with the assay set as spatial (raw counts), clusters as predictors, and offset set as UMI number per spot (nCount_Spatial), to generate the NEBULA object. The design matrix supplied to the NEBULA command included NMF cluster (one hot encoded; the first cluster with fewer than five DEGs from the preliminary differential expression analysis and are set as the reference cluster) and CARD deconvolution proportions for all cell types. Entries in the NEBULA results that failed to converge (convergence indicator ≤ −20) are discarded. A gene was deemed differentially expressed in a cluster if that cluster was associated with the highest statistically significant log fold-change coefficient.

#### Differential gene expression for Denisenko Visium data set and downstream analysis

Preliminary differential expression analysis for the integrated Denisenko Visium data set was performed using Seurat FindMarkers() function under a log fold-change.threshold parameter of 0.25 and min.pct parameter of 0.1, comparing spots from each cluster with all other spots (one vs. all).

NEBULA was used for the second round differential expression analysis. The integrated SeuratObject of the eight Denisenko samples were supplied to the NEBULA scToNeb() function, with the assay set as spatial (raw counts), clusters as predictors, and offset set as the UMI number per spot (nCount_Spatial), to generate the NEBULA object. The design matrix supplied to the NEBULA command included Seurat cluster (one hot encoded; the first cluster with fewer than five DEGs from the preliminary differential expression analysis and are set as the reference cluster) and CARD deconvolution proportions for all cell types. Entries in the NEBULA results that have failed to converge (convergence indicator ≤ −20) were discarded. A gene was deemed differentially expressed in a cluster if that cluster was associated with the highest statistically significant log fold-change coefficient.

To assess whether observed correlation structures exceeded expectations under spatial independence, we implemented permutation-based null correlation models at two levels:

1. Module score level spatial correlations. For each spatial sample, the spot-by-gene expression matrix (SCT for our analysis) and spot-level module scores are taken to compute the observed pairwise correlations between module scores using Spearman's. To generate a spatial-null distribution of each pairwise module versus module spatial correlation, we leave one module score−spot mapping untouched and permute randomly the other module score−spot mapping and compute the Spearman's correlation of the permuted module score pair for 1000 permutations. From each permuted module pair, we compute the empirical two-sided *P*-value for observed correlation. Within each sample, empirical *P*-values across module pairs were adjusted using the BH procedure to control the false-discovery rate (FDR).
2. Selected gene level spatial correlations. For each spatial sample, the spot-by-gene expression matrix (SCT for our analysis) for the specific gene sets (described above) is taken to compute the observed pairwise correlations between gene expression using Spearman's. Only genes present in all samples were included. To generate a spatial-null distribution of each pairwise gene versus gene spatial correlation, we leave one gene expression–spot mapping untouched and permute randomly the other gene–spot mapping and compute the Spearman's correlation of the permuted module score pair for 500 permutations. From each permuted module pair, we compute the empirical two-sided *P*-value for the observed correlation. Within each sample, empirical *P*-values across module pairs were adjusted using the BH procedure to control the FDR.

All permutation analyses were conducted independently per spatial sample. Correlations were computed across spatial spots using scaled gene expression values, and empirical *P*-values were derived from permutation-based null distributions.

After obtaining the DEG sets corresponding to each cluster, we isolated two immune gene programs (histocompatibility and immunoglobulin DEGs) and calculated the Spearman's correlation across sets of DEGs to quantify the degree to which upregulated cluster-specific gene sets interact. The results were visualized using a heatmap.

#### scRNA-seq feature selection, dimensionality reduction, and unsupervised clustering

Integration of Utah and Denisenko single-cell data sets (*N* = 15,578) was performed with scVI-tools v1.0.4, SCANPY v1.9.6, anndata 0.1.3. Preintegration feature selection of top 3000 highly variable genes was performed on merged raw count matrices using SCANPY with the flavor parameter set as Seurat v3 and the batch key parameter set as sample identifier (Wolf et al. 2018; Gayoso et al. 2022; Virshup et al. 2024). Additionally, the marker genes from 32 stromal subclusters from the Qian et al. (2020) pancancer blueprint were included as the features to integrate. Integration was achieved by training a scVI model with two layers, latent space dimension of 30, and negative binomial likelihood model and parameter batch key set as sample identifier. Clustering of the scVI (Gayoso et al. 2022) integrated single-cell data set was performed with the SCANPY pp.neighbors and tl.leiden functions with default parameters with the scVI learned latent representation, resulting in 19 total clusters (Supplemental Fig. S4A).

#### Differential gene expression using scVI integrated scRNA-seq data

Differential expression analysis for the integrated scRNA-seq data set was performed with scVI-tools differential expression function, using Leiden clusters as grouping variables (and by default conducts 1 cluster vs. all testing). The DEGs were filtered by lfc_mean > 2.0, bayes_factor > 3, non_zeros_proportion1 > 0.5, and non_zeros_proportion2 < 0.25.

Three clusters (clusters 0, 14, and 6) had fewer than 10 DEGs under such criteria. Four of the 19 Leiden clusters (cluster 5, 6, 8, 9) were identified as tumor/epithelial cells owing to their expression of canonical epithelial ovarian cancer markers, including *WFDC2* and *ELF3* (Supplemental Fig. S4B,D,E; Hellström et al. 2003; Xu et al. 2021). Clusters 12 and 16 were identified as mesothelial cells by marker gene expression of *CALB2*, *KRT8*, *KRT18*, and *WT1* (Olbrecht et al. 2021). Cluster 7 was identified as endothelial cells by expression of canonical marker genes *CLDN5*, *VWF*, and *CLEC14A* (Supplemental Fig. S4B,D,E; Qian et al. 2020; Olbrecht et al. 2021).

Seven of the 19 Leiden clusters were identified as immune cells: First, a T/NK cell cluster (2) expressing *NKG7* and *CD3D* was detected (Qian et al. 2020). Another cluster (10) expressing *FKBP51* and *RHOH* appeared as an additional T lymphocyte cluster (Chae et al. 2010; Tong et al. 2023). Cluster 15 expressed *IGHG1* and *IGHG4* and was denoted B/plasmablast. Cluster 3 showed expression of *CD68*, *MSR1*, and *MRC1* and was hence identified as macrophage. Cluster 17 expressed considerably less macrophage marker *CD83* and showed high expression of *CD14*; hence, we denoted it as a separate monocyte population. Cluster 18 lacked *CD68* but expressed high levels of CD83 as well as pDC markers *LILRA4* and *CXCR3* and hence was identified as a pDC population (Supplemental Fig. S4B,D,E; Qian et al. 2020).

Six of the 19 Leiden clusters were identified as fibroblast or myofibroblasts: Cluster 13 showed high expression of *LUM*, *DCN*, and *DPT* and was denoted as fibroblast (DPT) population. Clusters 11 and 16 showed expression of college subunits including *COL1A1*, *COL1A2*, and *COL3A1*, with cluster 16 additionally expressing *FN1* and *EBF1*, and hence were classified as fibroblast (COL1A1) and fibroblast (FN1), respectively. Clusters 0 and 1 had moderate expression of *DCN* and expressed moderate to high amounts of the *STAR* and *FOXL2* genes and were identified as two fibroblast (STAR) populations. Lastly, cluster 4 expressed the canonical myofibroblast marker genes *ACTA2* and *TAGLN* and hence was denoted as myofibroblasts (Supplemental Fig. S4B,D,E; Qian et al. 2020).

Upon examining the expression of marker gene sets published by Denisenko et al. (2024), we were able to recover most cell types (tumor/epithelial; mesothelial; endothelial; T cell; B cell; macrophage; fibroblast subpopulations 1, 2, 3, and 5; and myofibroblasts). The profile of Denisenko fibroblast 4, marked by expression of *CCL2*, did not match any distinct Leiden cluster in our integration. Nonetheless, the myofibroblast population (Leiden cluster 4) in our integration had moderate expression of *CCL2*, potentially indicating that some of the cells corresponding to the Denisenko fibroblast 4 populations may be clustered with myofibroblasts by scVI integration (Supplemental Fig. S4D).

### Functional enrichment analyses

Functional enrichment analysis comparing functional profile of clusters was performed with the compareCluster() and enrichGO() functions from the clusterProfiler (v4.8.3) package (Wu et al. 2021b), using NEBULA DEG-integrated Denisenko samples as the input and querying the GO Biological Processes database.

The functional enrichment results were visualized with the emapplot() function from the enrichplot (v1.20.3) package, with plotting layout set as “kk” (https://bioconductor.org/packages/enrichplot).

### Spot-level deconvolution of cell types

Spatially aware deconvolution was performed for each of the 11 spatially resolved samples using the CARD (v1.1) method (Ma and Zhou 2022).

The reference data set for CARD deconvolution runs was constructed by integrating five HGSC 10x Genomics single-cell data sets published by Denisenko et al. and seven additional HGSC 10x Genomics scRNA-seq data sets generated at HCI. Merged raw count matrices of the single-cell data sets, along with metadata specifying cell-type annotations and sample IDs, were used as reference input to CARD deconvolution.

### HGSC subtype assignment

Subtype assignment to samples was achieved with the *consensusOV* (v1.22.0) package using pseudobulked expression, generated by summing across raw count matrices across spots within each sample and, subsequently, library size-normalized and log-transformed (Chen et al. 2018). Molecular subtype classification was attempted using different methods implemented by the package: “consensusOV”, “Helland”, “Konecny”, and “Verhaak”.

### cell–cell communication analysis

Cell–cell communication analysis on the Denisenko Visium samples was performed with COMMOT (Cang et al. 2023). The Visium raw counts were normalized with SCANPY with default parameters and log-transformed. Initial filtering of potential ligand-receptor pairs was done with COMMOT's filter_lr_database function, using CellChat as the database and minimum_cell = 5 as the filtering criteria (Jin et al. 2021). Cell–cell interaction is inferred with COMMOT spatial_communication, with the interaction distance set as 250.

### cell2location analysis

The cell2location reference regression model is trained based on the integrated scRNA-seq data set (see for integration methods). Only cells corresponding to the same donor as the Utah spatial samples from integrated scRNA-seq were retained, and subclustering is applied to epithelial cell clusters identified in the annotation (Supplemental Fig. S5) and then filtered using the gene and cell filtering parameters as suggested by the official documentation (cell_count_cutoff = 5, cell_percentage_cutoff = 0.03, nonz_mean_cutoff = 1.12). The estimated expression profile was exported from the trained reference model and passed to the cell2location function with each Visium sample, using N_cells_per_location = 5 and detection_alpha = 200. The fifth percentile estimate for cell-type abundance computed by cell2location in each spot was plotted.

## Data access

All raw sequencing data generated in this study have been submitted to the NCBI database of Genotypes and Phenotypes (dbGaP; https://dbgap.ncbi.nlm.nih.gov/home/) under accession number phs002262.v3.p1. The R and Python code to reproduce all preprocessing, analyses, and figures in this paper is available at GitHub (https://github.com/wli51/Inter_patient_heterogeneity_HGSOC_spatial_codes), Zenodo (https://doi.org/10.5281/zenodo.18810839), and as Supplemental Code.

## Supplemental Material

Supplement 1

Supplement 2
