# Supplementary material for: Characterizing intra- and intertumor heterogeneity in ovarian high-grade serous carcinoma subtypes using single-cell and spatial transcriptomics
Source: Genome Res. 2026 Oct;36(10):2011–26. doi: 10.1101/gr.281433.125 (PMC13629689; doi:10.1101/gr.281433.125)
Supplement: Supplementary file 1 [file Supplemental_Material.pdf]

Supplemental Materials

Characterizing intra- and inter-tumor heterogeneity in ovarian high-grade serous carcinoma subtypes using single-cell and spatial transcriptomics

Weishan Li, Laurie Grieshober, Jason Gertz, Adriana Ivich, Jennifer A. Doherty, Casey S. Greene*, Stephanie C. Hicks*

*Co-Corresponding authors: Casey S. Greene (casey.s.greene@cuanschutz.edu) and Stephanie C. Hicks (shicks19@jhu.edu)

Contents

1. **Supplemental Tables S1-S4**
2. **Supplemental Figures S1-S16**

QC-summary					
Sample	pre-QC n-cells	post-QC n-cells	pre-QC n-genes	post-QC n-genes	source
16030X2	7123	3469	36601	22537	HCI at Utah
16030X3	1533	1102	36601	19931	HCI at Utah
16030X4	6546	3260	36601	23782	HCI at Utah
19459X1	4727	3272	36601	27498	HCI at Utah
19595X1	2595	1151	36601	26289	HCI at Utah
19833X1	2916	2536	36601	25857	HCI at Utah
19833X2	3402	2788	36601	23917	HCI at Utah

Table S1: Summary of Utah HCI scRNA-seq samples before and after quality control (QC) filtering.

Sample ID	Source	Seuqencing Method	cOV Molecular Subtype
2B	HCI at Utah	10x Visium	DIF
3C	HCI at Utah	10x Visium	DIF
4D	HCI at Utah	10x Visium	DIF
SP1	Denisenko et. al	10x Visium	DIF
SP2	Denisenko et. al	10x Visium	MES
SP3	Denisenko et. al	10x Visium	MES
SP4	Denisenko et. al	10x Visium	DIF
SP5	Denisenko et. al	10x Visium	IMR
SP6	Denisenko et. al	10x Visium	IMR
SP7	Denisenko et. al	10x Visium	DIF
SP8	Denisenko et. al	10x Visium	MES

Table S2: Sample-level summary of the of the Utah HGSC Visium samples. For each 10x Genomics Visium capture area (rows), the table includes the Sample ID, the source or where the tissue was obtained from, the experimental protocol used, and the predicted cOV molecular subtype.

		SP1			SP4			SP7		
Pathway	Ligand-Receptor	mean	1st quartile	3rd quartile	mean	1st quartile	3rd quartile	mean	1st quartile	3rd quartile
MK	MDK.ITGA6_ITGB1	2.76	0.03	4.22	1.21	0.00	0.25	1.26	0.00	0.13
	MDK.LRP1	9.89	3.10	13.66	8.37	0.30	13.06	8.39	0.00	11.54
	MDK.NCL	8.10	1.48	10.98	15.56	6.40	22.11	11.23	0.16	15.64
	MDK.SDC1	7.50	0.69	10.66	2.52	0.00	2.56	2.09	0.00	0.94
	MDK.SDC2	2.62	0.06	3.35	3.73	0.00	5.66	3.01	0.00	2.92
	MDK.SDC4	3.67	0.08	5.23	6.21	0.12	10.16	11.86	0.11	16.06
MIF	MIF.ACKR3	2.54	0.00	3.57	0.76	0.00	0.00	0.94	0.00	0.00
	MIF.CD74_CD44	1.70	0.00	1.63	2.82	0.00	1.90	6.73	0.00	9.46
	MIF.CD74_CXCR4	5.08	0.00	7.93	5.03	0.00	5.81	0.80	0.00	0.00
SPP1	SPP1.CD44	1.46	0.00	0.21	0.79	0.00	0.00	8.23	0.00	9.94
	SPP1.ITGA8_ITGB1	0.03	0.00	0.00	0.01	0.00	0.00	0.13	0.00	0.00
	SPP1.ITGA9_ITGB1	0.65	0.00	0.00	0.35	0.00	0.00	0.20	0.00	0.00
	SPP1.ITGAV_ITGB1	2.00	0.00	2.08	0.66	0.00	0.00	1.57	0.00	0.35
	SPP1.ITGAV_ITGB5	0.85	0.00	0.01	0.43	0.00	0.00	1.65	0.00	0.24
	SPP1.ITGAV_ITGB6	0.08	0.00	0.00	0.06	0.00	0.00	0.73	0.00	0.00
SEMA3	SEMA3A.NRP1_PLXNA1	0.16	0.00	0.00	0.05	0.00	0.00	0.21	0.00	0.00
	SEMA3A.NRP1_PLXNA2	0.08	0.00	0.00	0.04	0.00	0.00	0.10	0.00	0.00
	SEMA3A.NRP1_PLXNA4	0.06	0.00	0.00	0.01	0.00	0.00	0.01	0.00	0.00
	SEMA3B.NRP1_PLXNA1	0.39	0.00	0.00	0.14	0.00	0.00	0.36	0.00	0.00
	SEMA3B.NRP1_PLXNA2	0.21	0.00	0.00	0.13	0.00	0.00	0.13	0.00	0.00
	SEMA3B.NRP1_PLXNA4	0.13	0.00	0.00	0.03	0.00	0.00	0.02	0.00	0.00
	SEMA3B.NRP2_PLXNA1	0.57	0.00	0.01	0.96	0.00	0.00	0.86	0.00	0.00
	SEMA3B.NRP2_PLXNA2	0.23	0.00	0.00	0.57	0.00	0.00	0.30	0.00	0.00
	SEMA3B.NRP2_PLXNA4	0.23	0.00	0.00	0.06	0.00	0.00	0.03	0.00	0.00
	SEMA3C.NRP1_NRP2_PLXND1	0.17	0.00	0.00	0.10	0.00	0.00	0.28	0.00	0.00
	SEMA3C.NRP1_PLXNA1	0.20	0.00	0.00	0.21	0.00	0.00	0.24	0.00	0.00
	SEMA3C.NRP1_PLXNA2	0.09	0.00	0.00	0.13	0.00	0.00	0.12	0.00	0.00
	SEMA3C.NRP1_PLXNA4	0.06	0.00	0.00	0.03	0.00	0.00	0.01	0.00	0.00
	SEMA3C.NRP2_PLXNA1	0.20	0.00	0.00	0.63	0.00	0.00	0.61	0.00	0.00
	SEMA3C.NRP2_PLXNA2	0.09	0.00	0.00	0.43	0.00	0.00	0.27	0.00	0.00
	SEMA3C.NRP2_PLXNA4	0.10	0.00	0.00	0.08	0.00	0.00	0.03	0.00	0.00
	SEMA3C.PLXND1	1.55	0.00	0.00	2.15	0.00	0.21	3.11	0.00	3.00
	SEMA3D.NRP1_PLXNA1	0.03	0.00	0.00	0.01	0.00	0.00	0.00	0.00	0.00
	SEMA3D.NRP1_PLXNA2	0.03	0.00	0.00	0.01	0.00	0.00	0.00	0.00	0.00
	SEMA3D.NRP1_PLXNA4	0.01	0.00	0.00	0.00	0.00	0.00	0.00	0.00	0.00
	SEMA3D.NRP2_PLXNA1	0.04	0.00	0.00	0.02	0.00	0.00	0.01	0.00	0.00
	SEMA3D.NRP2_PLXNA2	0.02	0.00	0.00	0.00	0.00	0.00	0.00	0.00	0.00
	SEMA3D.NRP2_PLXNA4	0.02	0.00	0.00	0.00	0.00	0.00	0.00	0.00	0.00
	SEMA3G.NRP2_PLXNA1	0.26	0.00	0.00	0.05	0.00	0.00	0.04	0.00	0.00
	SEMA3G.NRP2_PLXNA2	0.11	0.00	0.00	0.04	0.00	0.00	0.02	0.00	0.00
	SEMA3G.NRP2_PLXNA4	0.13	0.00	0.00	0.01	0.00	0.00	0.01	0.00	0.00
ncWNT	WNT11.FZD1	0.28	0.00	0.00	0.11	0.00	0.00	0.20	0.00	0.00
	WNT11.FZD2	0.32	0.00	0.00	0.09	0.00	0.00	0.10	0.00	0.00
	WNT11.FZD3	0.35	0.00	0.00	0.14	0.00	0.00	0.14	0.00	0.00
	WNT11.FZD4	0.24	0.00	0.00	0.11	0.00	0.00	0.19	0.00	0.00
	WNT11.FZD7	0.42	0.00	0.00	0.12	0.00	0.00	0.11	0.00	0.00
	WNT11.FZD8	0.14	0.00	0.00	0.01	0.00	0.00	0.06	0.00	0.00
	WNT5A.FZD1	0.46	0.00	0.00	1.08	0.00	0.00	0.37	0.00	0.00
	WNT5A.FZD2	0.58	0.00	0.00	1.26	0.00	0.00	0.25	0.00	0.00
	WNT5A.FZD3	0.60	0.00	0.00	1.93	0.00	0.11	0.37	0.00	0.00
	WNT5A.FZD4	0.38	0.00	0.00	1.45	0.00	0.00	0.41	0.00	0.00
	WNT5A.FZD7	0.77	0.00	0.00	1.20	0.00	0.00	0.27	0.00	0.00
	WNT5A.FZD8	0.25	0.00	0.00	0.09	0.00	0.00	0.15	0.00	0.00
	WNT5A.MCAM	0.93	0.00	0.00	0.53	0.00	0.00	0.30	0.00	0.00
PTN	PTN.NCL	5.74	0.00	8.25	0.40	0.00	0.00	0.20	0.00	0.00
	PTN.SDC1	4.47	0.00	6.98	0.16	0.00	0.00	0.08	0.00	0.00
	PTN.SDC2	1.71	0.00	2.35	0.17	0.00	0.00	0.08	0.00	0.00
	PTN.SDC3	1.53	0.00	1.52	0.14	0.00	0.00	0.09	0.00	0.00
	PTN.SDC4	2.32	0.00	3.33	0.25	0.00	0.00	0.19	0.00	0.00
PDGF	PDGFA.PDGFRA	0.18	0.00	0.00	0.62	0.00	0.00	0.40	0.00	0.00
	PDGFA.PDGFRB	0.38	0.00	0.00	0.39	0.00	0.00	0.52	0.00	0.00
	PDGFC.PDGFRA	1.30	0.00	0.00	3.70	0.00	1.23	0.81	0.00	0.00
	PDGFD.PDGFRB	0.48	0.00	0.00	0.37	0.00	0.00	0.22	0.00	0.00
COMPLEMENT	C3.C3AR1	0.57	0.00	0.00	0.57	0.00	0.00	0.78	0.00	0.00
	C3.ITGAM_ITGB2	0.07	0.00	0.00	0.05	0.00	0.00	0.31	0.00	0.00
	C3.ITGAX_ITGB2	0.10	0.00	0.00	0.02	0.00	0.00	0.49	0.00	0.00
CXCL	CXCL10.ACKR1	0.44	0.00	0.00	0.01	0.00	0.00	0.10	0.00	0.00
	CXCL12.ACKR3	0.45	0.00	0.00	0.22	0.00	0.00	0.27	0.00	0.00
	CXCL12.CXCR4	1.35	0.00	0.00	0.84	0.00	0.00	0.33	0.00	0.00
	CXCL2.ACKR1	0.07	0.00	0.00	0.13	0.00	0.00	0.22	0.00	0.00
	CXCL8.ACKR1	0.01	0.00	0.00	0.18	0.00	0.00	0.47	0.00	0.00

Table S3: Summary table for ligand-receptor outgoing signal strength from top (active) signaling pathways for DIF Visium samples (SP1, SP4, SP7), organized by Pathway. Values represent the mean, first quartile, and third quartile communication scores for each ligand–receptor pair within the indicated pathway, as inferred from cell–COMMOT Jin et al. 2021 cell communication analysis. Higher values (length of highlight lenghts) indicate stronger predicted signaling activity from the sender cell population toward all potential receiver populations in the dataset.

QC-summary					
Sample	pre-QC n-cells	post-QC n-cells	pre-QC n-genes	post-QC n-genes	source
Y2	328	328	19915	13377	Denisenko et. al
Y3	6384	6384	19915	17247	Denisenko et. al
Y5	7588	7588	19915	17618	Denisenko et. al
MJ10	426	426	19915	15248	Denisenko et. al
MJ11	1956	1956	19915	17591	Denisenko et. al

Table S4: Summary of from Denisenko et al. scRNA-Seq dataset before and after quality control (QC) filtering.

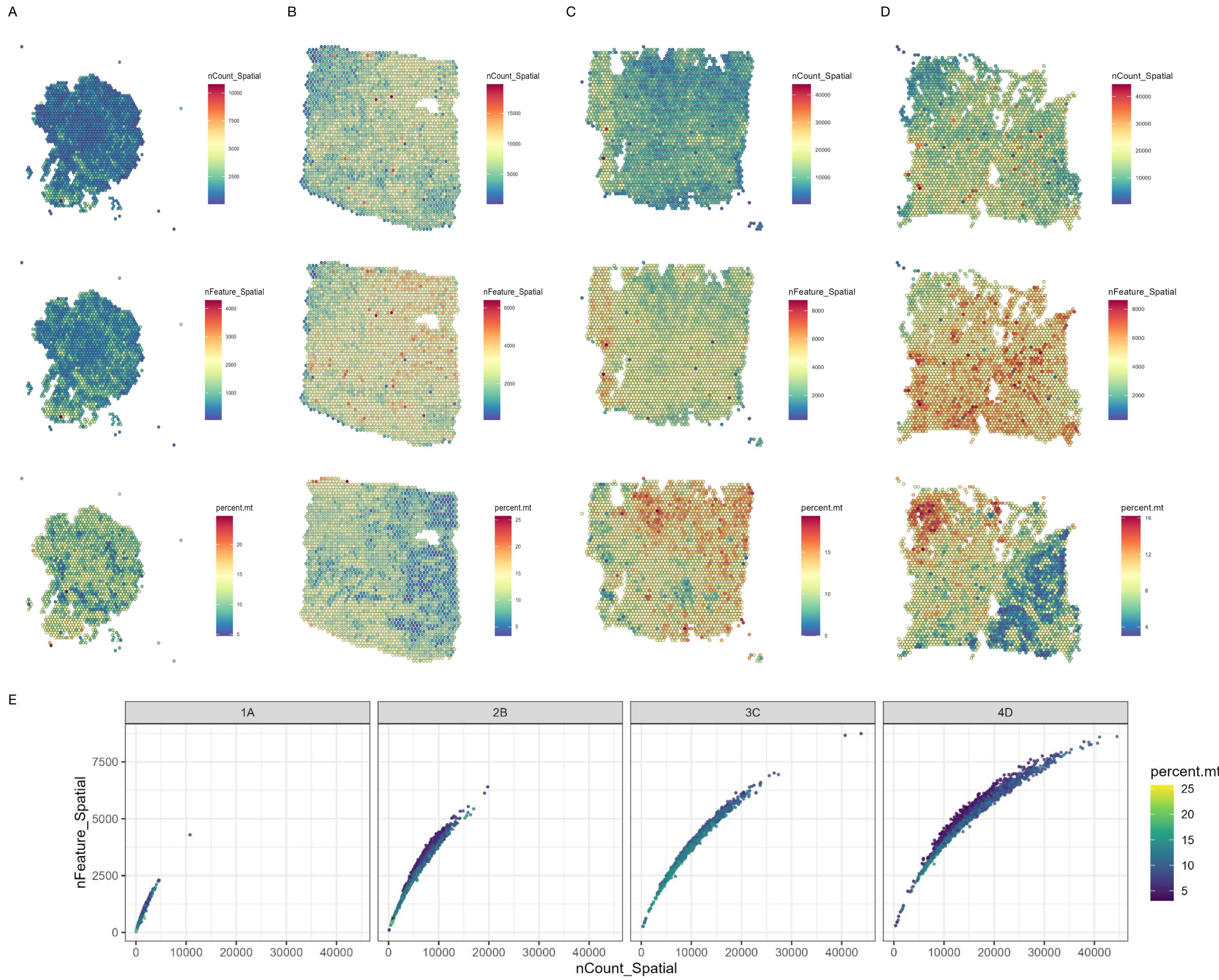

Figure S1: Quality control metrics for N=4 tissue sections. Distribution of total UMIs per capture area for tumor sample 16030X2. Capture area 1A was removed from downstream analysis due to low total number of UMI. **(A-D)** Spatial visualization of spot-wise UMI count (top), expressed gene count (middle) and mitochondrial gene concentration (bottom) across the four capture areas **(E)** Summary scatter plot for the same quality control statistic across all capture areas

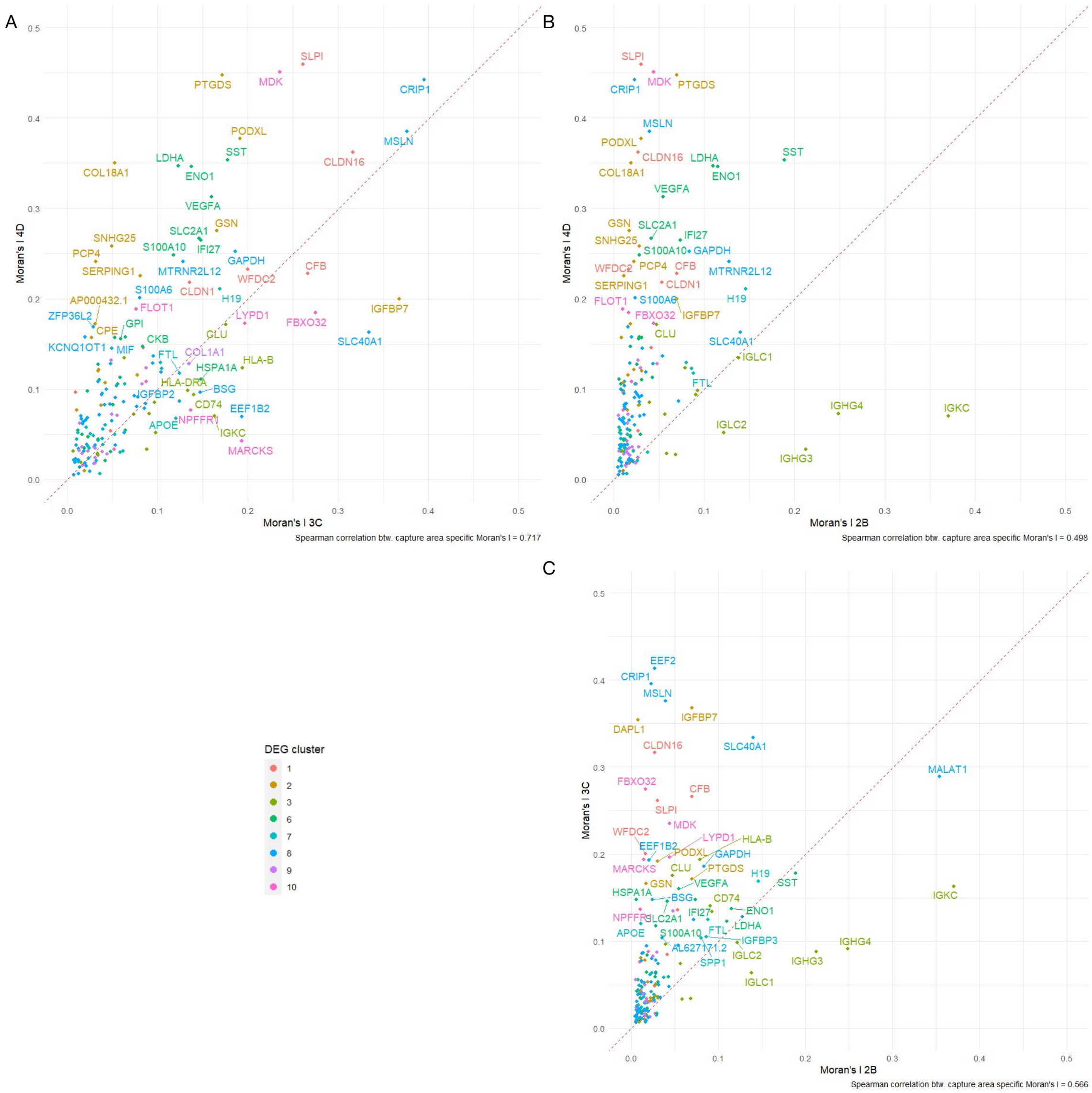

Figure S2: HGSC identify different spatially variable genes. (**A-C**) Pairwise scatterplots between pairs of N=3 capture areas with the spatially variable genes detected using Moran's I statistic, gene name labels are shown only for genes with absolute Moran's I exceeding 0.1 in either sample. The genes names and scatter points were colored in accordance to the NMF cluster from which the gene is identified as DEG.

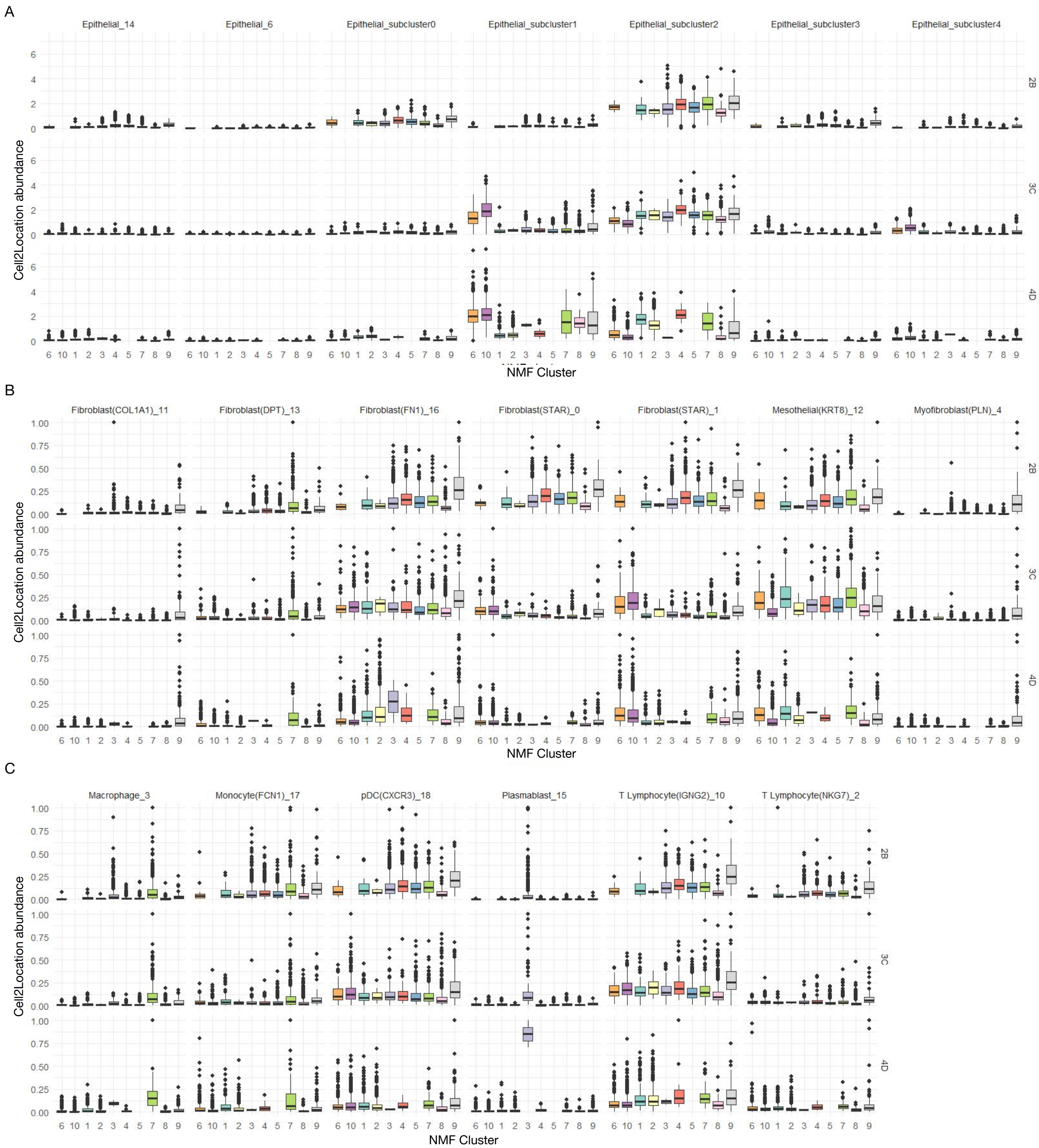

Figure S3: (Caption next page.)

Figure S3: Cell2location epithelial cell abundance. Summary statistics of 5th percentile cell-type abundance predictions by *Cell2location* mapping of clustered single cell data from **(A)** Epithelial clusters, **(B)** Stromal clusters and **(C)** Immune clusters. The spatial-domain formed by high-abundance Epithelial-subcluster1 subpopulations in 3C and 4D corresponds well with the NMF clustering scheme (cluster 6 and 10) from **Fig. 2**. Fibroblast(STAR) populations also seemed to be exclusively abundant in clusters 6 and 10 while higher Fibroblast(FN1) proportions are associated with other NMF clusters.

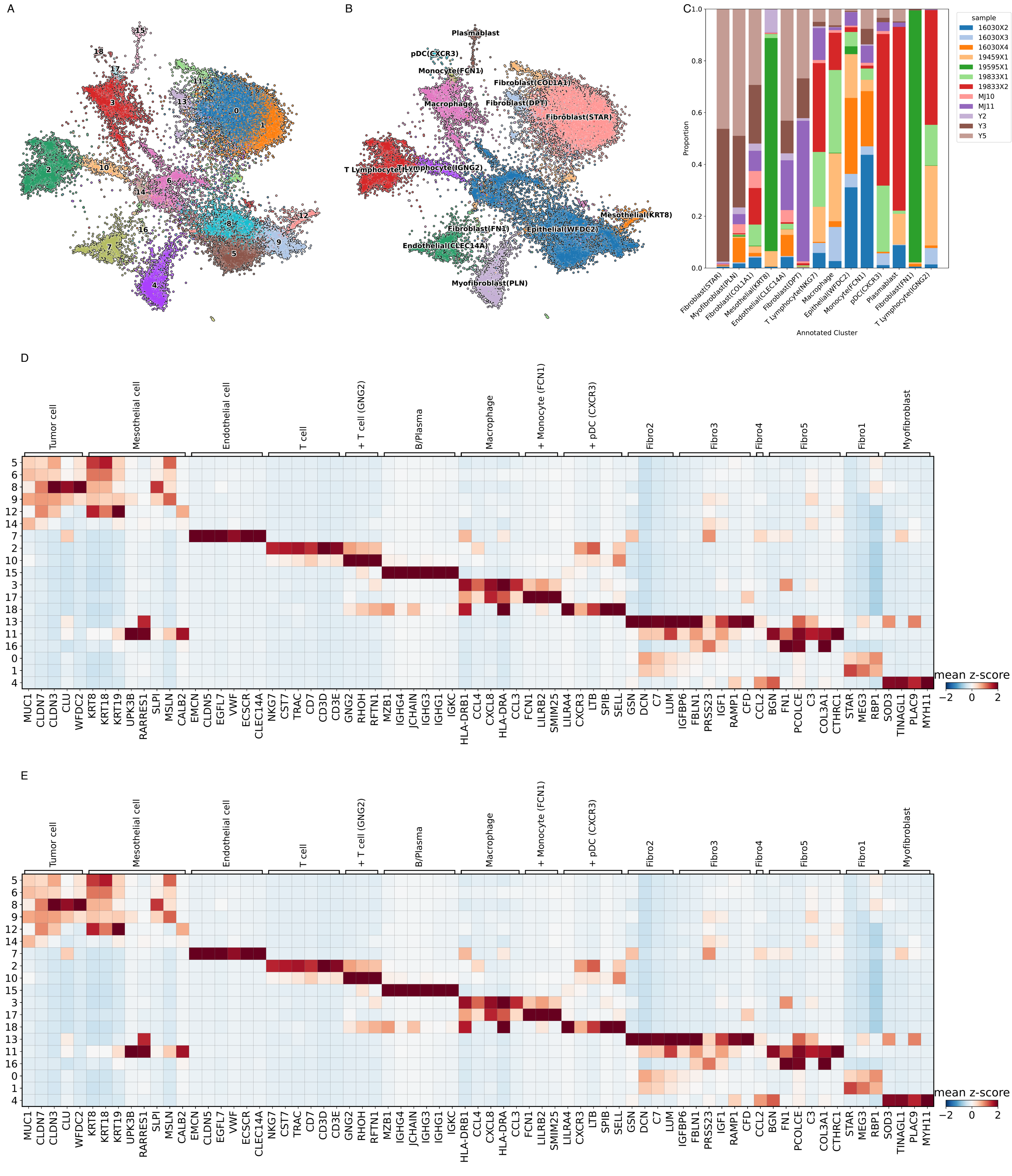

Figure S4: (Caption next page.)

Figure S4: Annotated single cell dataset summary. UMAP projection of annotated integrated single cell dataset that is used as reference in *CARD* deconvolution, colored by **(A)** leiden cluster number and **(B)** annotated cell type label. **(C)** Relative abundance of samples across all integrated clusters. **(D)** Matrixplot illustration of marker genes corresponding to each of the annotated cell types. **(E)** Matrixplot illustration of the markers corresponding to the cell type annotation reported by Denisenko et. al. Note that the scVI integration and annotation differed from the Denisenko reference annotation primarily in fibroblast subpopulations. The Denisenko Fibro5 (*FN1*, *COL3A1*) seemed to best correspond to the scVI Fibroblast (*COL1A1*), and Denisenko Fibro2 (*RBP1*, *DCN*) best corresponded scVI Fibroblast (*DPT*), Alistair Fibro3 (*RAMP1*, *CFD*) best corresponded to scVI Fibroblast (*RAMP1*), and Alistair Myofibroblast corresponded to scVI Myofibroblast (*PLN*; *ACTA2*). There seemed to be a lack of correspondence between Alistair Fibro1 and Fibro4 with any of the scVI fibroblast types.

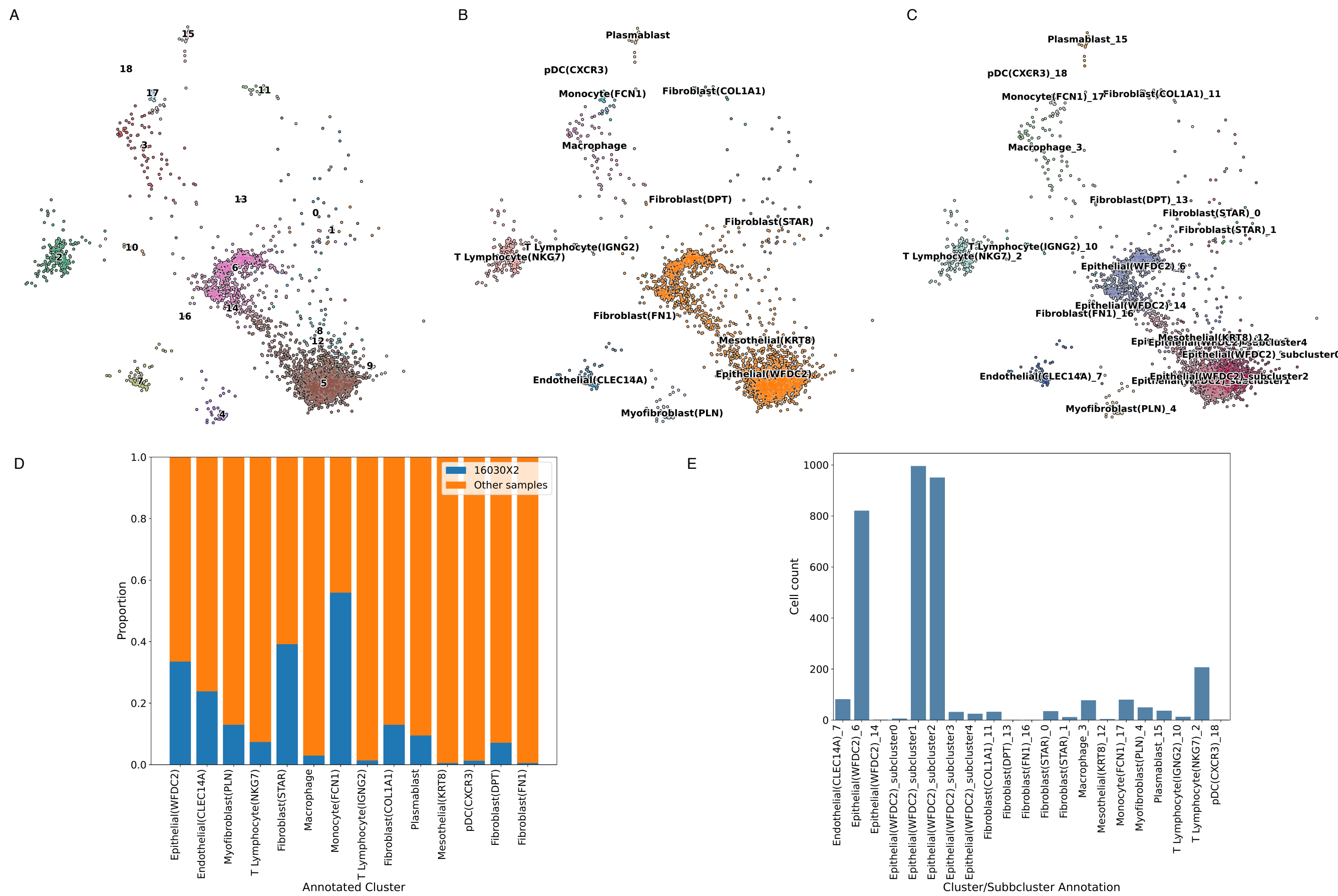

Figure S5: Annotated scRNA-seq data Utah subsetted to only sample 16030X2. UMAP visualization of 16030X2 single cell landscape, colored by **(A)** leiden cluster number derived from same the integration step, also presented in **Supplemental Fig. S4** **(B)** annotated cell type label from the same integration step, and **(C)** annotated cell type label, with the epithelial clusters further subclustered. **(D)** Relative abundance of 16030X2 cells across all integration clusters. **(E)** Absolute count of 16030X2 cells across the sample specific clusters and subclusters.

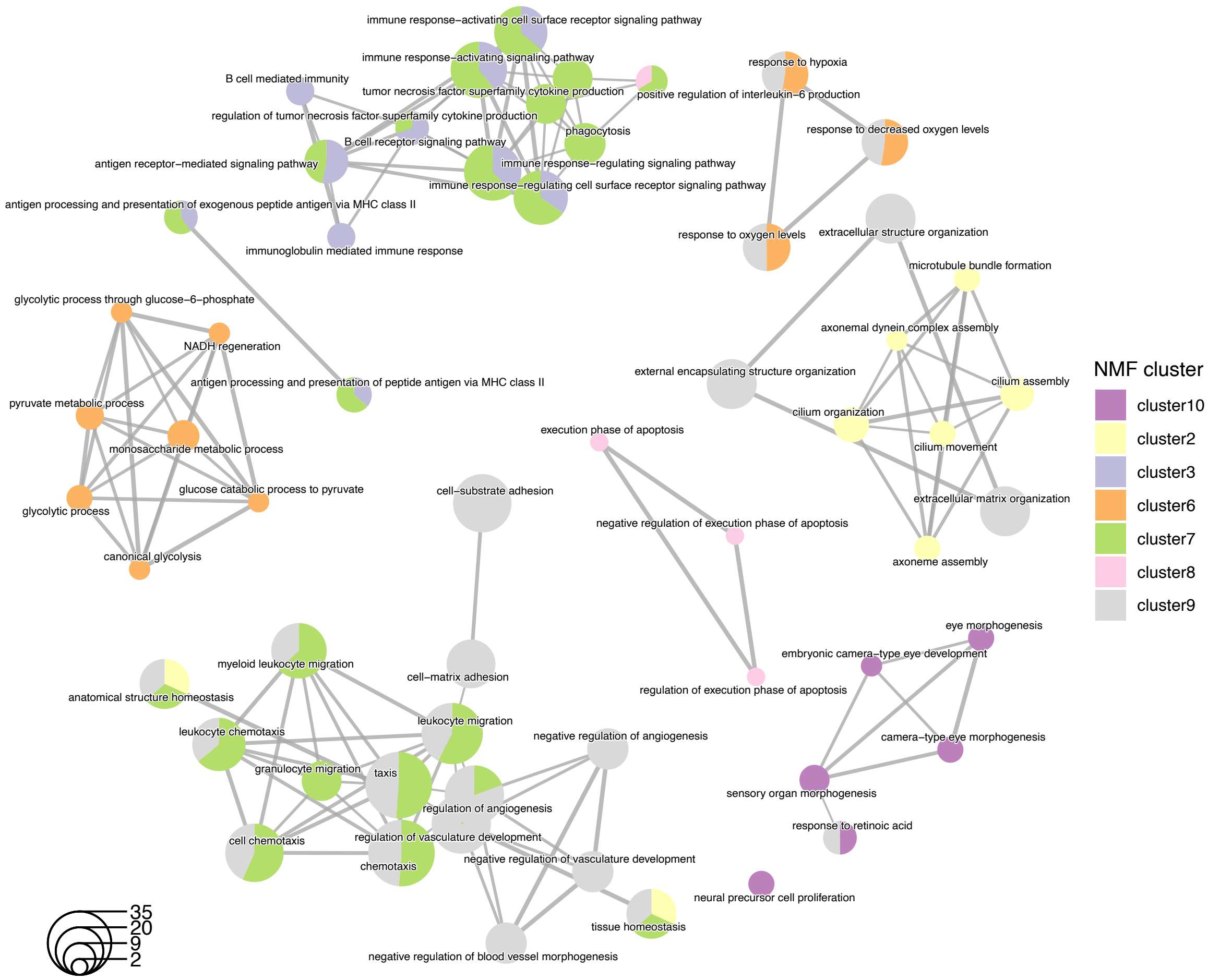

Figure S6: Functional enrichment network of domain-specific DEGs across NMF spatial domains across Utah spatial samples. DEGs identified for each NMF spatial domain are visualized as an enrichment map network. Nodes represent enriched GO terms (scaled by gene count), and edges indicate shared gene overlap between terms. Node colors correspond to NMF domains (**Fig. 2**. Top panel is the simplified version with only group terms visualized, the bottom is the full detailed version with node specific labels. NMF cluster 10 (predominantly present in capture areas 3C and 4D) formed a tightly connected module enriched for epithelial and morphogenetic programs, including eye/camera-type morphogenesis, sensory organ development, and cilium organization, suggesting structured epithelial differentiation states. Cluster 9, also enriched in 3C/4D, was associated with extracellular matrix organization and angiogenesis-related processes. Notably, clusters 7 and 9 showed partial functional connectivity through terms related to vascular development and leukocyte migration, consistent with macrophage/CAF enrichment and stromal–immune interplay. In contrast, cluster 3 (primarily localized to 2B) formed a distinct immune-centered module enriched for antigen processing and presentation, B cell activation, and immune response–activating signaling pathways. Cluster 6 showed isolated enrichment for metabolic and glycolytic processes, while cluster 8 was primarily associated with apoptotic regulation.

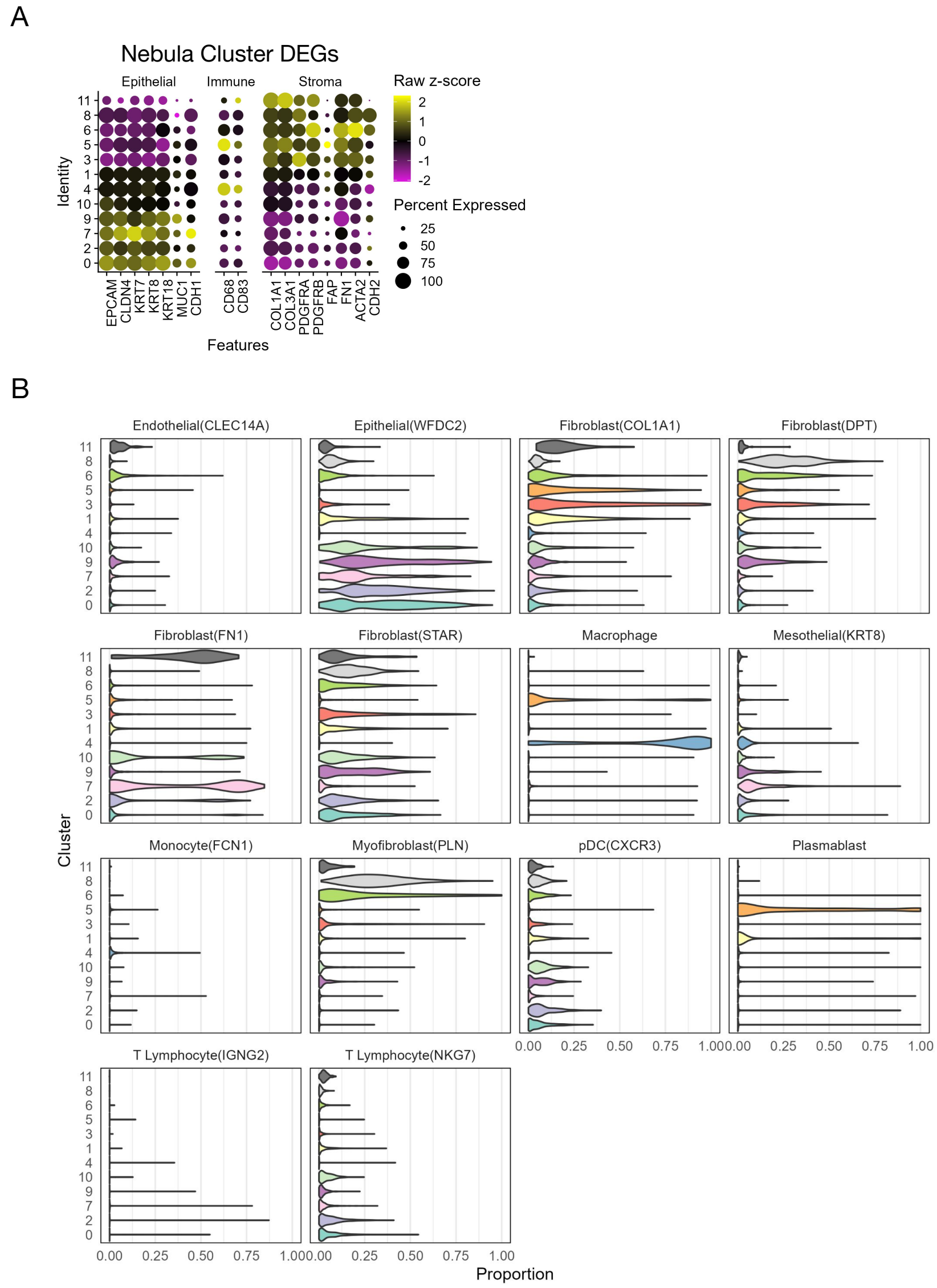

Figure S7: Canonical epithelial vs mesenchymal marker expression in the integrated dataset. **(A)** Dot plot showing expression of epithelial and mesenchymal markers. We found that clusters 0, 2, 7, 9 and 10 expressed higher epithelial markers and clusters 3, 5, 6, 8, 11 express higher mesenchymal markers.**(B)** Violin plot showing deconvolution proportions comparing epitheliat-dominant clusters (0, 2, 7, 9, 10) with fibroblast-dominant clusters (3, 5, 6, 8, 11).

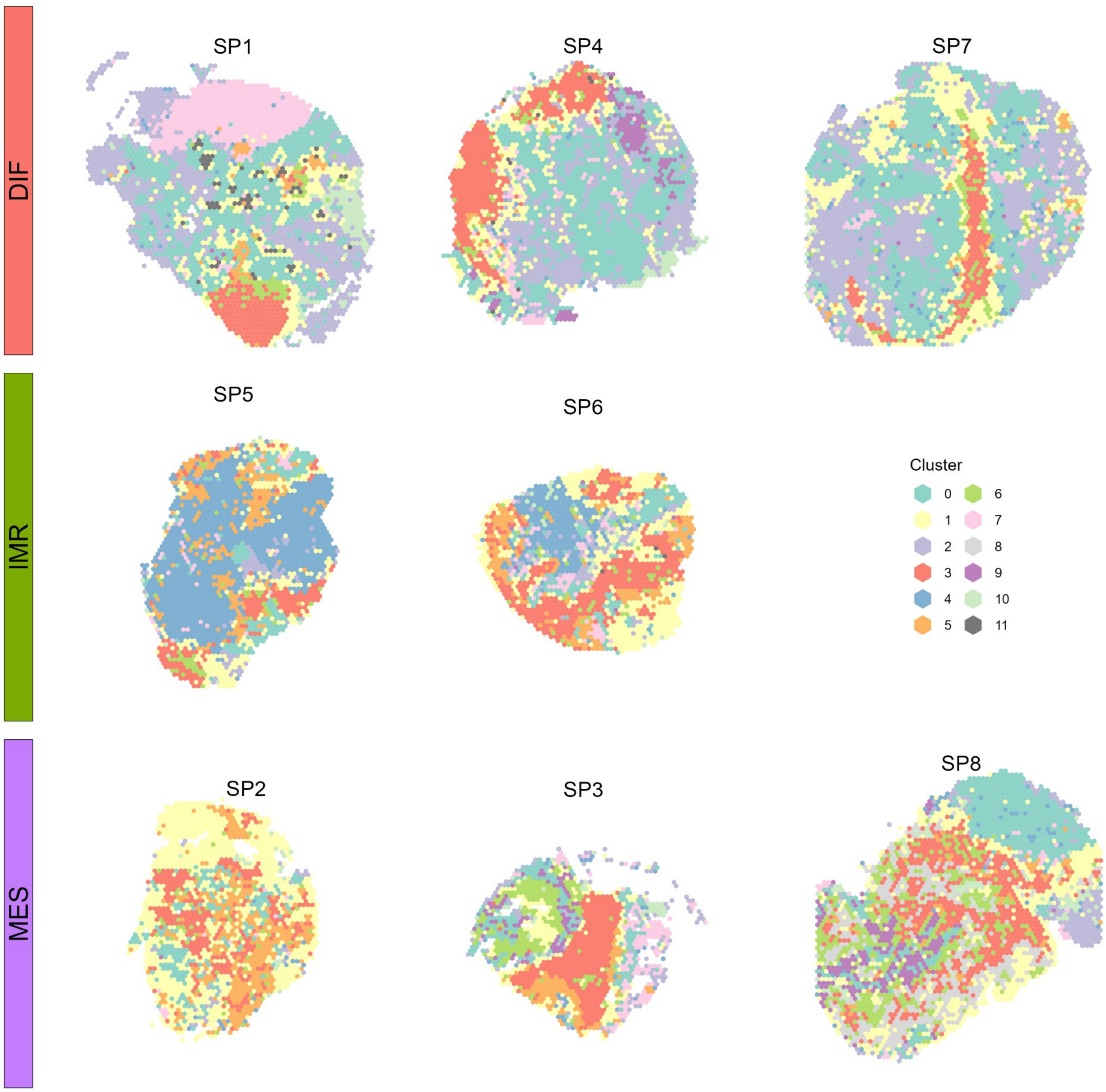

Figure S8: Integrated clustering in space. Clustering scheme in space for the integration of the Denisenko samples. In total 12 clusters are identified. Notably, cluster 1 (yellow) occurred almost exclusively between epithelial-dominant (teal, pink, blue-gray) and fibroblast-dominant clusters (red, orange, green, light gray).

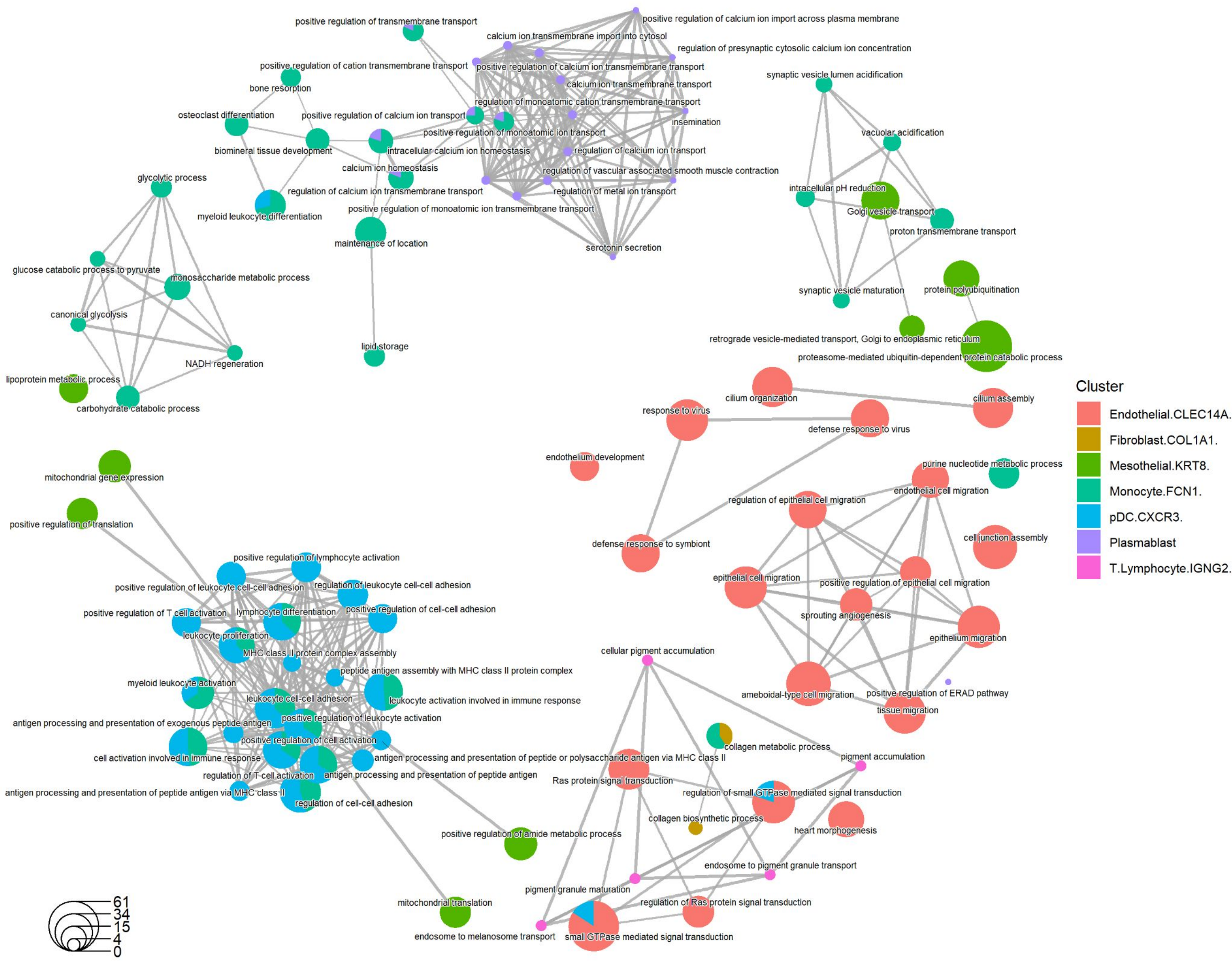

Figure S9: Gene concept network plot showing the GO (biological pathway) results based on genes whose expression is positively associated with the estimated proportion of cell types from spot-level deconvolution, colored by cell types. *KRT8*-expressing mesothelial cells were found to be enriched in functions such as protein polyubiquitination, while *COL1A1* expressing fibroblasts were functionally enriched in collagen biosynthetic and metabolic process. The functional annotations associated with immune cells except pDC are mostly descriptive of subcellular activities: endosome to melanosome transport and cellular pigment accumulation are the top biological themes for T lymphocytes (potentially reflective of cell proliferation in spots with higher T cell abundance); ER to cytosol transportation and cellular response to unfolded proteins are the top themes associated with spots of high plasmablasts abundance; cross-membrane transport and ion homeostasis are the top theme for monocytes.

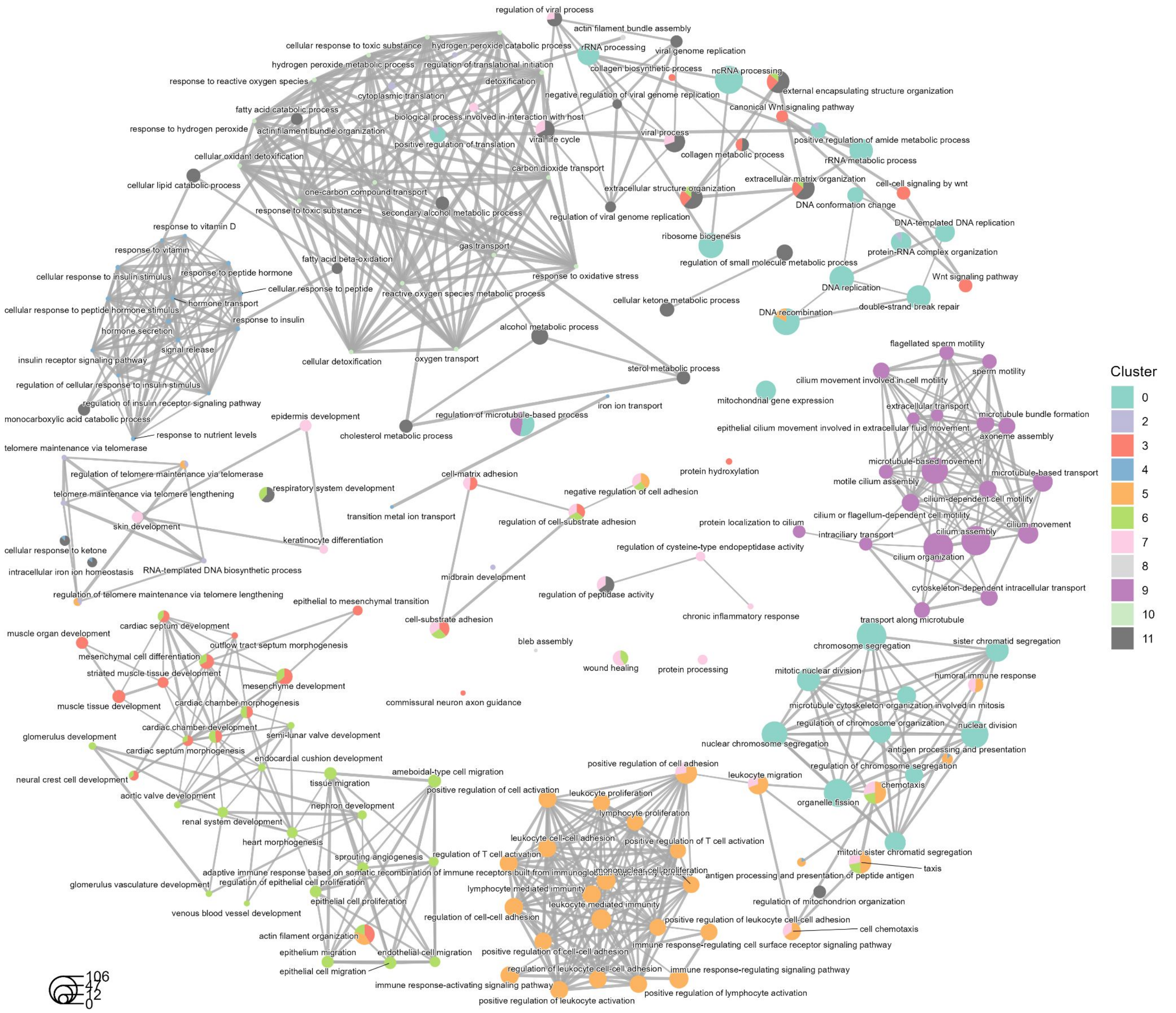

Figure S10: Full Gene Ontology (GO) enrichment network across clusters in integrated spatial samples. Comprehensive GO enrichment network constructed from cluster-specific differentially expressed genes. The functional GO (Gene Ontology) enrichment results obtained using *clusterProfiler* Wu et al. 2021, from spatial domain-specific, upregulated DEGs sets. Each node represents an enriched biological process, and edges indicate shared gene overlap between terms. Node color corresponds to cluster identity as defined in the integrated analysis. The network resolves into multiple topologically distinct modules representing immune/inflammatory, proliferative/cell cycle, ciliary/motility, developmental/morphogenesis, oxidative stress/metabolic, and RNA processing programs. The dense intra-module connectivity and limited inter-module overlap demonstrate substantial functional diversification across clusters. The simplified representation shown in the main figure was derived from this full network by collapsing individual GO terms into dominant thematic modules.

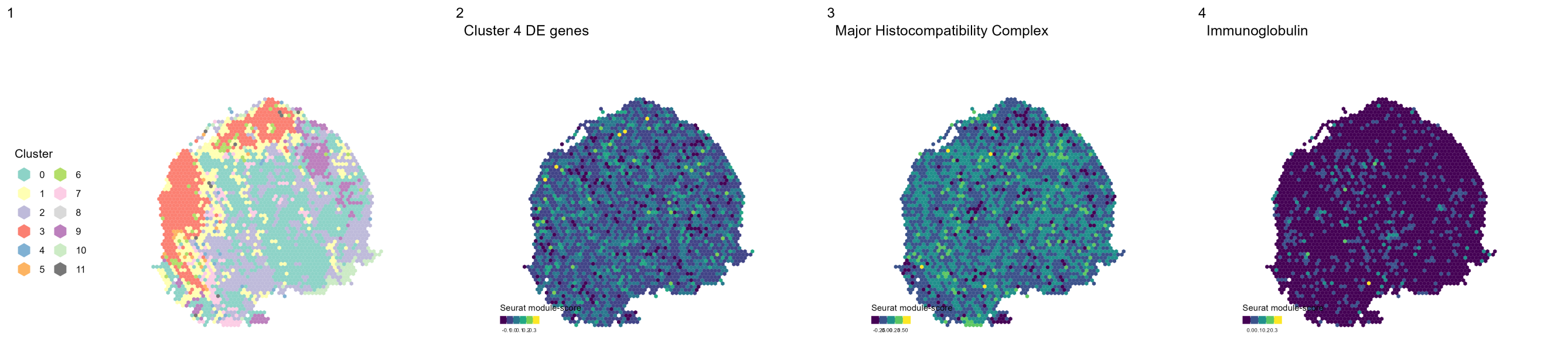

Figure S11: Denisenko et al. sample SP4 (DIF) spatial domain. Immunoglobulin and major histocompatibility complex genes scores visualization for SP4, which is not included in **Fig. 5**. Compared to the other DIF samples, SP4 displays less co-expression of MHC and IG modules in space.

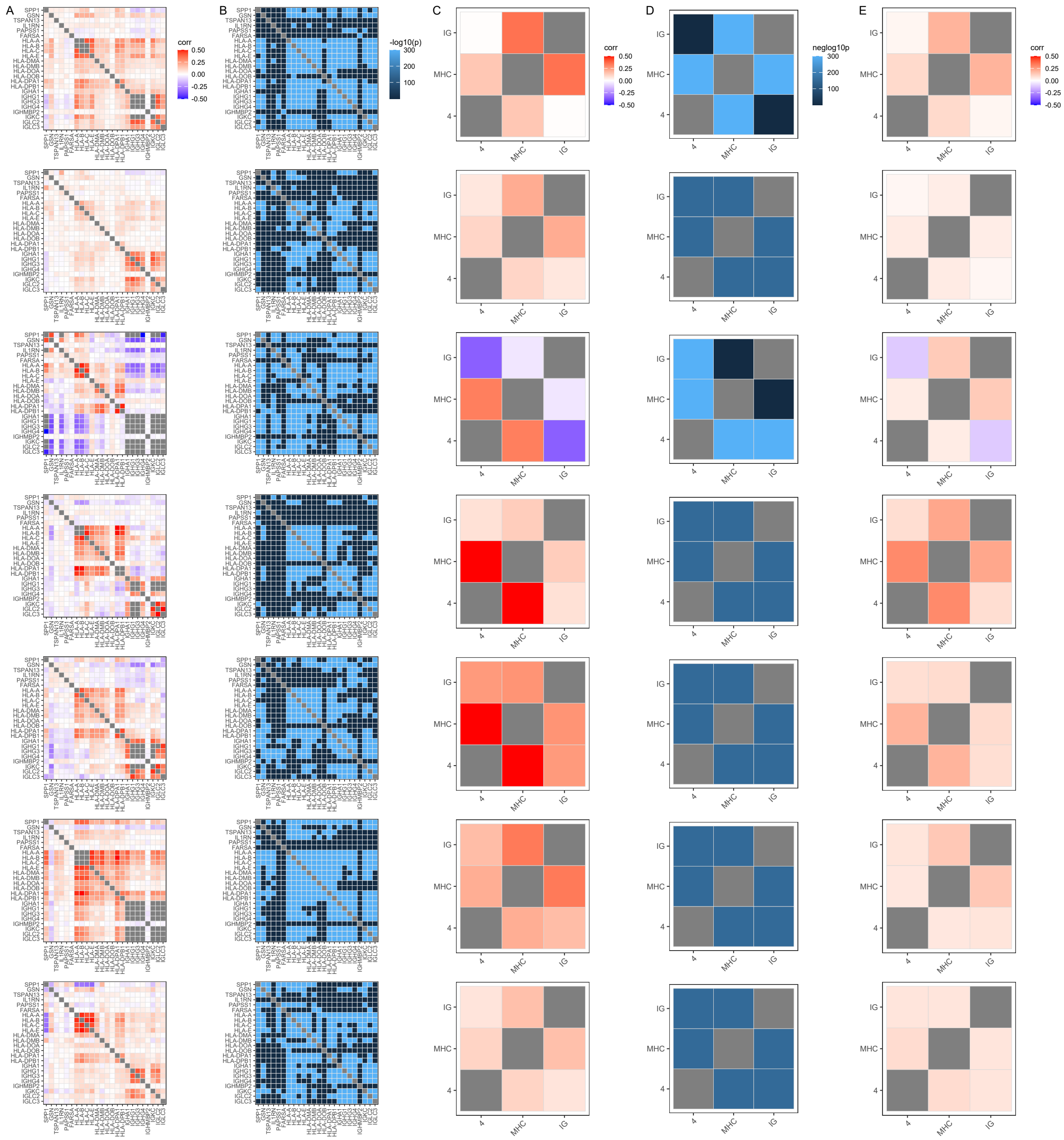

Figure S12: Spatial null testing and composition-adjusted partial correlation analysis of immune gene programs. (**A**) Observed Spearman correlations at gene level (**B**) Empirical correlation p values against permuted spots. (**C**) Observed Spearman correlations at module level (**D**) Empirical correlation p values against permuted spots. (**E**) Module level partial correlations from module scores residualized on deconvolution-estimated cell-type fractions (adjust gene program association for composition). Composition adjustment attenuated several associations, including the IG–MHC anti-correlation in IMR samples, but did not uniformly abolish inter-program structure. These results suggest that while immune cell density contributes to observed spatial relationships, subtype-specific spatial organization of immune gene programs is not fully explained by compositional variations.

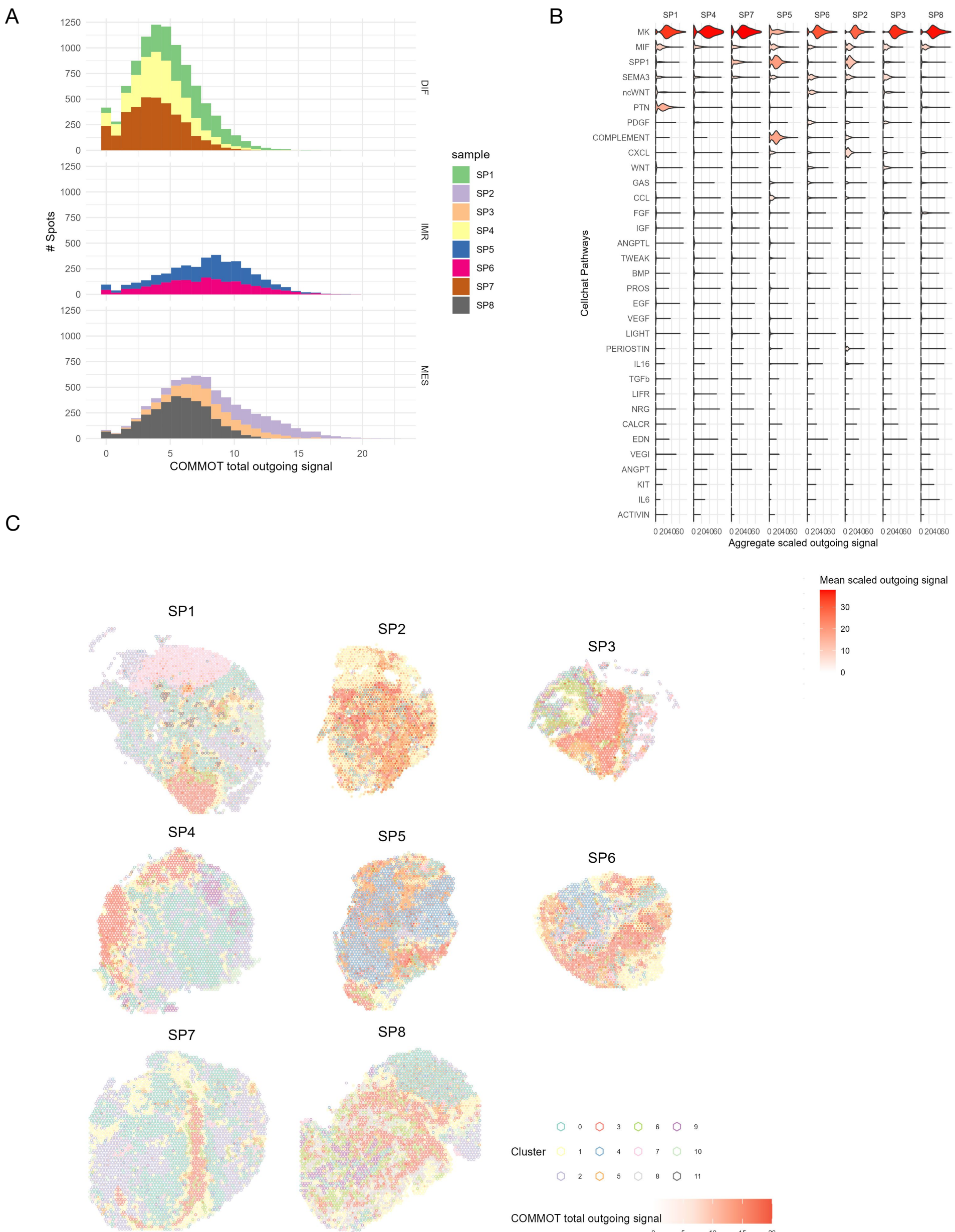

Figure S13: COMMOT ligand-receptor total signaling summary. **(A)** Histogram indicating distribution of spot total outgoing signals in the N=8 Denisenko Visium samples, grouped by molecular subtype. The magnitude and distribution of total outgoing signal is similar between samples of the same subtype. In general, the IMR samples are more active compared to the MES and DIF samples. **(B)** Violin plot summarizing the distribution of scaled outgoing signaling in each pathway across all samples. **(C)** Total signaling activity in space. Signaling activity is represented with fill color, while cluster is represented as boundary line of spots. A substantial amount of signaling occurs near the boundary of clusters.

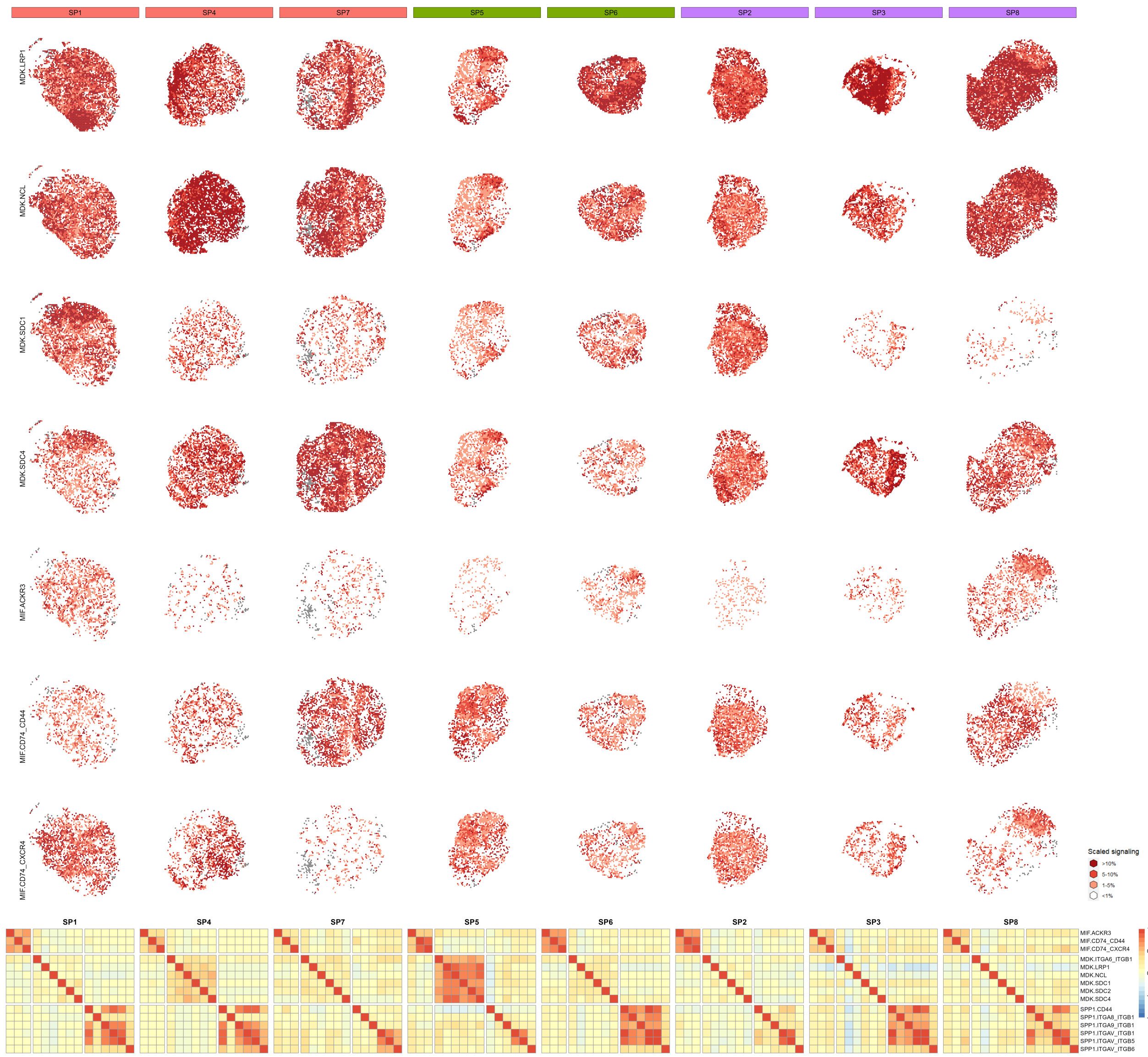

Figure S14: COMMOT ligand-receptor signaling correlation. [Spatial plots, top] Scaled signaling activities in select ligand receptor pairs. Each sample is described by one column, indicated by the column label. Each selected ligand receptor pair is represented by the same row. [Heatmap, bottom] Spearman correlation of ligand receptor pairs from the top 3 most active pathways (MIF, MK, SPP1). The correlation structure appeared to be highly sample specific and blacked subtype specific patterns, except for the MES specific anti-correlation between MDK-LRP1 signaling with other MK, MIF and SPP1 pathway ligand receptor pairs (heatmap SP3, SP8). Notably, ligand receptor activities from the MK pathway clearly colocalize to different cluster/spatial domains of the tumor tissue in SP1, SP7, SP3 and SP8, corresponding to the lack of inter-pathway correlation in the corresponding heatmaps.

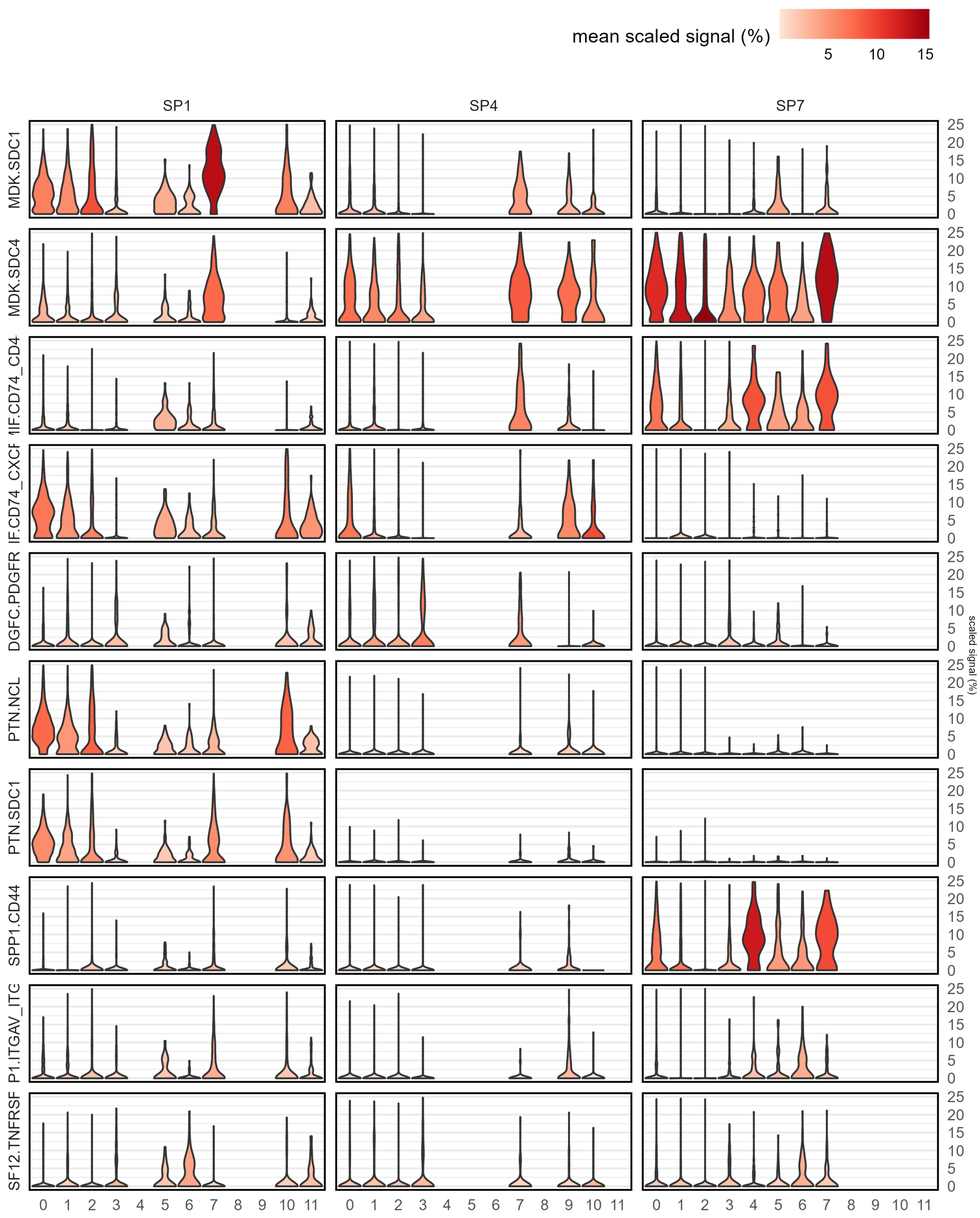

Figure S15: COMMOT select ligand-receptor pair signaling strength summary for DIF samples. The violin plot represents the distribution of scaled outgoing signal strength. The fill of each violin corresponds to the mean scaled outgoing signal strength in spatial domain of sample. Domains with fewer than 50 spots per sample omitted.

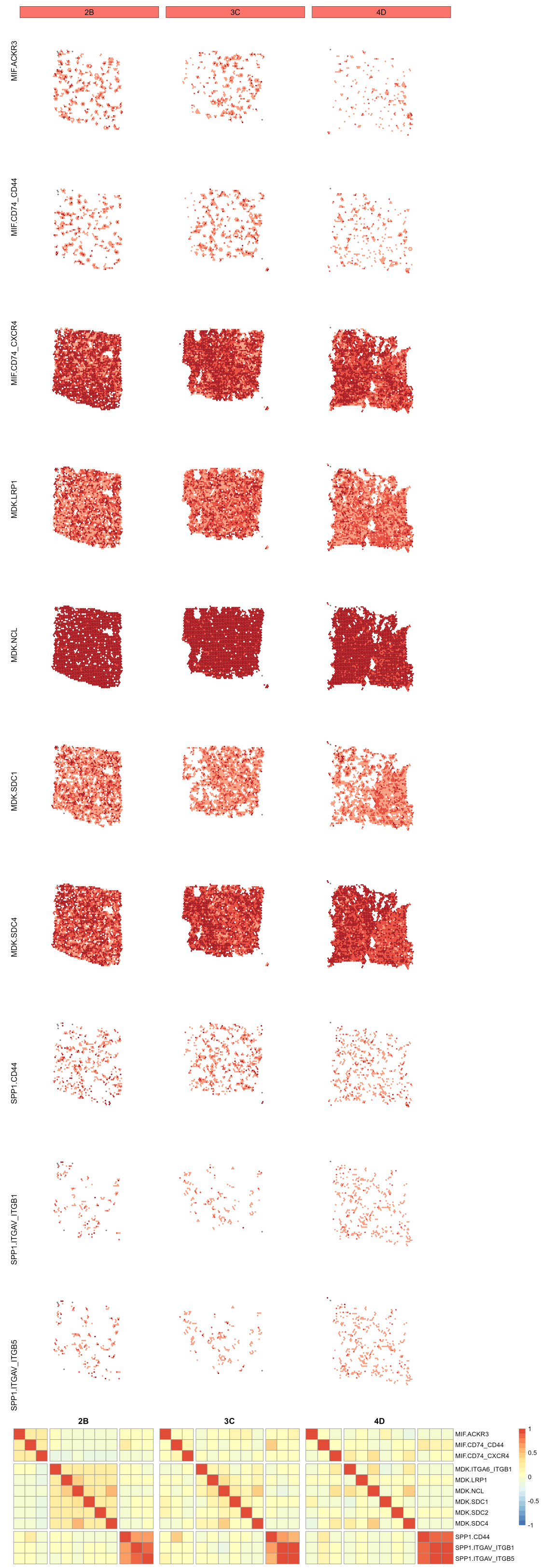

Figure S16: (Caption next page.)

Figure S16: COMMOT ligand-receptor signaling correlation in Utah tissue slices. [Spatial plots, top] Scaled ligand–receptor (LR) signaling activities computed using the identical pipeline applied to the public cohort. Each column represents one Visium tissue slice (2B, 3C, 4D) derived from the same tumor and classified as the same bulk DIF subtype **Figure 2**. Each row corresponds to a selected LR pair from the three most active pathways (MIF, MK, SPP1), shown in the same order as in the public dataset figure to enable direct comparison. [Heatmap, bottom] Spearman correlation of ligand receptor pairs from the top 3 most active pathways (MIF, MK, SPP1). In contrast to the marked inter-sample heterogeneity observed across the eight Denisko et. al tumors **Figure S14**, the three Utah slices demonstrate highly concordant spatial activity patterns and spatial correlation structure. In contrast to the marked inter-sample heterogeneity observed across the eight public tumors, the Utah sections demonstrated highly concordant spatial signaling architectures. The spatial distribution of MK-, MIF-, and SPP1-associated ligand-receptor activities was reproducible across all three slices, with similar intensity gradients and spatial domains. Consistent with this observation, correlation analysis revealed a preserved intra-pathway structure across the Utah sections.
